# WHO 2010 Guidelines for Prevention of Mother-to-Child HIV Transmission in Zimbabwe: Modeling Clinical Outcomes in Infants and Mothers

**DOI:** 10.1371/journal.pone.0020224

**Published:** 2011-06-02

**Authors:** Andrea L. Ciaranello, Freddy Perez, Matthews Maruva, Jennifer Chu, Barbara Engelsmann, Jo Keatinge, Rochelle P. Walensky, Angela Mushavi, Rumbidzai Mugwagwa, Francois Dabis, Kenneth A. Freedberg, for the CEPAC-International Investigators

**Affiliations:** 1 Division of Infectious Diseases, Massachusetts General Hospital, Boston, Massachusetts, United States of America; 2 INSERM U897 Africa Team, Institut de Santé Publique, d'Epidémiologie et de Développement (ISPED), Université Victor Segalen Bordeaux 2, Bordeaux, France; 3 Pan American Health Organization, HIV/AIDS Unit, Washington, D.C., United States of America; 4 Elizabeth Glaser Pediatric AIDS Foundation: Zimbabwe Country Office, Harare, Zimbabwe; 5 Division of General Medicine, Massachusetts General Hospital, Boston, Massachusetts, United States of America; 6 Organization of Public Health Interventions and Development (OPHID) Trust, Harare, Zimbabwe; 7 Division of Infectious Disease, Brigham and Women's Hospital, Boston, Massachusetts, United States of America; 8 Center for AIDS Research, Harvard Medical School, Boston, Massachusetts, United States of America; 9 Ministry of Health and Child Welfare, Harare, Zimbabwe; University of Cape Town, South Africa

## Abstract

**Background:**

The Zimbabwean national prevention of mother-to-child HIV transmission (PMTCT) program provided primarily single-dose nevirapine (sdNVP) from 2002–2009 and is currently replacing sdNVP with more effective antiretroviral (ARV) regimens.

**Methods:**

Published HIV and PMTCT models, with local trial and programmatic data, were used to simulate a cohort of HIV-infected, pregnant/breastfeeding women in Zimbabwe (mean age 24.0 years, mean CD4 451 cells/µL). We compared five PMTCT regimens at a fixed level of PMTCT medication uptake: 1) no **antenatal** ARVs (comparator); 2) sdNVP; 3) WHO 2010 guidelines using “Option A” (zidovudine during pregnancy/infant NVP during breastfeeding for women without advanced HIV disease; lifelong 3-drug antiretroviral therapy (ART) for women with advanced disease); 4) WHO “Option B” (ART during pregnancy/breastfeeding without advanced disease; lifelong ART with advanced disease); and 5) “Option B+:” lifelong ART for all pregnant/breastfeeding, HIV-infected women. Pediatric (4–6 week and 18-month infection risk, 2-year survival) and maternal (2- and 5-year survival, life expectancy from delivery) outcomes were projected.

**Results:**

Eighteen-month pediatric infection risks ranged from 25.8% (no **antenatal** ARVs) to 10.9% (Options B/B+). Although maternal short-term outcomes (2- and 5-year survival) varied only slightly by regimen, maternal life expectancy was reduced after receipt of sdNVP (13.8 years) or Option B (13.9 years) compared to **no antenatal ARVs (14.0 years)**, Option A (14.0 years), or Option B+ (14.5 years).

**Conclusions:**

Replacement of sdNVP with currently recommended regimens for PMTCT (WHO Options A, B, or B+) is necessary to reduce infant HIV infection risk in Zimbabwe. The planned transition to Option A may also improve both pediatric and maternal outcomes.

## Introduction

Antiretroviral drugs (ARVs) are highly effective for the prevention of mother-to-child HIV transmission (PMTCT). Without ARV prophylaxis, the risk of transmission by 18 months of age ranges from 25–40% in breastfeeding populations [1]–[3]. However, recent trials have demonstrated that several ARV regimens, administered to mothers and/or infants during pregnancy and breastfeeding, can reduce transmission to 1–8% at 6–12 months of age [4]–[10]. The World Health Organization (WHO) now recommends three-drug antiretroviral therapy (ART) for all pregnant women with CD4 cells <350/µL or clinical Stage 3–4 disease. For those with less advanced disease, two options are recommended. “Option A” includes short-course zidovudine during pregnancy and extended infant nevirapine (NVP) prophylaxis throughout breastfeeding. “Option B” includes maternal 3-drug ART during pregnancy and breastfeeding, with cessation after weaning [11]. Select PMTCT programs in sub-Saharan Africa are implementing Option B, and an “Option B+” has also been proposed (lifelong ART for all pregnant, HIV-infected women, regardless of CD4 cell count or disease stage) [12]. However, most programs in sub-Saharan Africa plan to implement the WHO 2010 guidelines by selecting Option A [12].

In 2009, only 53% of pregnant women identified as HIV-infected worldwide received any ARVs for PMTCT, resulting in approximately 370,000 new infant infections [13]. Many of these women received the ARV regimens previously recommended as a “minimum” intervention by WHO: a single dose of nevirapine (sdNVP) to a pregnant woman in labor and her infant after birth [13], [14]. Although inexpensive and relatively easy to administer, sdNVP is less effective than currently recommended regimens (18-month transmission risks: 15–25%) [15]–[17] and can lead to drug-resistant virus that complicates later therapy for both mothers and infected infants [18], [19].

Zimbabwe is a low-income country where prolonged breastfeeding is the norm [20], [21]. The Zimbabwean Ministry of Health and Child Welfare (MOHCW) has provided sdNVP through the national PMTCT program since 2002, as one of the earliest PMTCT programs in Africa [22]. In 2006–2007, the MOHCW, with the Elizabeth Glaser Pediatric AIDS Foundation and the Organization of Public Health Interventions and Development Trust, demonstrated the feasibility of a pilot program providing the antenatal/intrapartum component of the WHO Option A regimen [23]. Our objective was to use simulation modeling to evaluate the potential benefits to both infants and mothers of replacing sdNVP with WHO 2010-recommended regimens on a national scale in Zimbabwe.

## Methods

### Analytic overview

**Figure 1:**
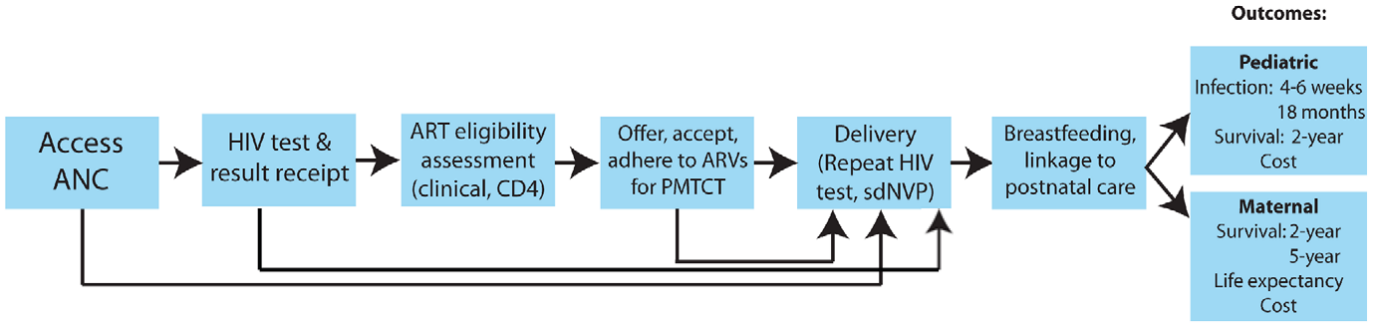
“Cascade” of PMTCT and postnatal HIV care. Opportunities to maximize the effectiveness of PMTCT interventions may be lost at each step in the pathway. ANC: antenatal care, ARVs: antiretroviral drugs, ART: antiretroviral therapy, sdNVP: single-dose nevirapine.

We linked two published computer simulation models to project clinical outcomes of five PMTCT strategies in Zimbabwe. First, a model of mother-to-child transmission (MTCT) during pregnancy and delivery [24] was modified to incorporate each step of the “cascade” of PMTCT-related care, from first presentation at antenatal care (ANC) through 18 months postpartum (Figure 1). Second, the Cost-effectiveness of Preventing AIDS Complications (CEPAC)-International model of adult HIV infection [25], [26] was used to project clinical outcomes for women following pregnancy, and was expanded to simulate infant outcomes from birth through the first two years of life. The models were linked by using CEPAC results as MTCT model inputs (Text S1). Outcomes of the linked models included risk of infant HIV infection at 4–6 weeks and 18 months of age and 2-year pediatric survival, as well as maternal 2-year survival, maternal 5-year survival, and maternal life expectancy after delivery. Additional details of model structure, data inputs, sensitivity analyses, and results are presented in the Appendix (Text S1).

### Population and PMTCT strategies evaluated

We simulated a population of pregnant and breastfeeding women in Zimbabwe who were HIV-infected at the time of conception [27]. For women identified as HIV-infected during ANC, five PMTCT strategies were evaluated (Text S1): 1) A “no antenatal ARVs” strategy, for reference comparison; 2) a single-dose of nevirapine (sdNVP) administered to laboring mothers and infants within 3 days of birth, reflecting the 2002–2009 national Zimbabwe PMTCT program and 2006 WHO “minimum” guidelines for resource-constrained settings [14]; 3) WHO 2010 “Option A:” short-course zidovudine during pregnancy and extended infant nevirapine during breastfeeding for women with CD4 >350/µL and no evidence of WHO stage 3–4 disease, with lifelong ART for women with advanced disease [11]; 4) WHO 2010 “Option B:” ART through pregnancy and breastfeeding regardless of CD4 or disease stage, with continuation after weaning for women with advanced disease [11]; and 5) the “Option B+” under consideration in select locations: lifelong ART for all pregnant, HIV-infected women, for comparison [12], [28]. ART-eligible women who linked to HIV-related healthcare after delivery were assumed to receive ART for their own health in all strategies (Text S1). Mothers were assumed to breastfeed their infants for a median duration of 18 months, based on Zimbabwean data [21]. Throughout the manuscript, the term “ARV” refers to any single or dual antiretroviral drug regimen used for PMTCT, while “ART” refers only to three-drug combination therapy (regardless if used for PMTCT or for therapy of maternal disease).

### Model structure

#### MTCT model

The MTCT model is a previously-published decision-analytic simulation of a cohort of pregnant women from conception through delivery [24] (TreeAgePro 2010 software, Williamstown, MA). The model structure was expanded to include key steps in antenatal care, as well as linkage to postnatal maternal and pediatric care (Figure 1 and Text S1). Probabilities of HIV transmission and maternal and infant death before and during delivery were stratified by the severity of maternal HIV infection (ART “eligible,” defined as WHO stage 3–4 disease or CD4 350/<µL [11]; “not eligible;” or deceased) and by maternal receipt of postnatal HIV care and ART.

#### CEPAC adult model

The CEPAC-International model is a first-order Monte Carlo simulation of HIV infection, in which patients are simulated individually from model entry through death. Details of model structure and validation have been published previously [25], [26], [29] and are further described in the Appendix (Text S1). In brief, disease progression is characterized by a sequence of monthly transitions between health states; these include acute opportunistic and other infections prevalent in southern Africa, chronic HIV infection, and death. The model records all clinical events during each patient's lifetime. A cohort of ten million women is simulated to produce stable estimates of outcomes (Text S1).

In the CEPAC adult model, current CD4 count, opportunistic infection prophylaxis, and history or absence of previous opportunistic infections determine the monthly risk of opportunistic infections and HIV-related death (Table 1). HIV RNA suppression with effective ART leads CD4 counts to increase, reducing the risks for opportunistic infections and death. Before initiation of ART or after virologic failure on ART, CD4 counts decline at a rate determined by current RNA level; this is accompanied by increased risks of opportunistic infections and death. After planned interruption of suppressive ART at the time of weaning (Option B only), CD4 counts decline more rapidly based on data from ART interruption and PMTCT trials [30]–[32].

**Table 1:** Selected model input parameters for a simulation model of strategies to prevent mother-to-child transmission of HIV in Zimbabwe: Maternal data.

Variable	Base Case Value	Range for sensitivity analyses	Data sources
**Baseline maternal cohort characteristics**			
Age (mean (SD), in years)	24.0 (5)	20–30	MOHCW [27]
HIV prevalence	16%		MOCHW [27]
HIV incidence	0.96%/year		MOHCW [39]
Mortality during pregnancy	0.7%	0–2%	MOHCW [27]
Live birth	95.7–98%	95–99%	MOHCW [27]
Among HIV-infected women at first ANC visit:			
CD4 count (mean (SD), cells/µL)	451 (50)		ZVITAMBO trial [21] (SD: assumption)
ART eligible	275 (50)		ZVITAMBO trial [21]
Not ART eligible	550 (50)		ZVITAMBO trial [21]
Incident infection in pregnancy	664 (50)		MACS [40]
Initial HIV RNA (mean copies/ml)	72,349		CTAC [41]
**Access to PMTCT “cascade”**			
Access to ANC	91%	80–100%	MOHCW [27]
HIV testing in ANC	87%	58–95%	MOHCW [23], [39]
Receipt of HIV test result in ANC	99%	71–99%	MOHCW [23], [39]
Sensitivity of clinical assessment of ART eligibility	36%	20–50%	MTCT-Plus Cohort [43]
Receipt of CD4 test result (Option A)	67%		[79]
Delivery in health care facility	69%	50–100%	MOHCW [23], [39]
If HIV status negative/unknown, test in labor	36%		MOHCW [23]
If HIV diagnosed in labor, receive sdNVP	80%		MOHCW [23]
**Postnatal HIV Care**			
Linkage to postnatal maternal care by 6 weeks postpartum	79%	51–100%	Average and range: [36], [44]–[48]
Loss to follow-up from postnatal maternal care	16% (year 1); 6%/year(years 2+)		[37], [80], [81]
**Maternal antiretroviral therapy**			
Efficacy (% HIV RNA suppression at 24 weeks)			
1^st^-line NVP/ZDV/3TC initiated in pregnancy	90%		[50]
1^st^-line NVP/d4T/3TC initiated post-partum			
With sdNVP exposure	85%	Difference between sdNVP exposed and unexposed: 0%–16%	OCTANE trial [49]Difference: [18], [69], [71]
Without sdNVP exposure**	90%		Difference assumed = 5% [50]
2nd-line LPV/r/TDF/FTC	72%		[82]
CD4 decline (6 months after ART interruption)	139 cells	75–139 cells/µL	[30]–[32]

#### CEPAC infant model

A first-order, Monte Carlo simulation model of infant HIV infection and survival was added to the CEPAC model. Modeled infants enter the postnatal model at birth. Based on events occurring before and during delivery in the MTCT model, infants are assigned one of three HIV categories (HIV-unexposed; HIV-exposed but uninfected; or HIV-infected) and three maternal disease categories (HIV-uninfected; HIV-infected and “ART eligible;” or HIV-infected and “ART non-eligible”). Over a two-year horizon, infants face a monthly probability of four key clinical events: 1) maternal HIV infection, if mother was previously uninfected, causing infants to transition from “unexposed” to “exposed-uninfected;” 2) maternal death, with risks derived from the adult CEPAC model as described above, after which infants are no longer at risk for HIV infection but are at higher risk of death due to orphanhood [33], [34]; **3)** infant HIV infection through breastfeeding, if infant was previously uninfected; and **4)** infant death from any cause. Risks of maternal death and of postnatal HIV transmission are stratified by maternal disease stage and ARV regimen, and risks of infant death are stratified by infant HIV exposure/infection status and receipt of ART if infected.

#### HIV/AIDS care and ART

For HIV-infected infants, a one-time probability of HIV diagnosis, linkage to HIV care, and ART initiation was modeled [13]. For mothers, care and ART were modeled in greater detail. A one-time probability of linkage to maternal HIV care was incorporated; this parameter reflects the probability that a post-partum mother will present to an HIV clinic for her own healthcare by six weeks postpartum. Women not linking to care within this period were assumed to present to HIV care upon later development of a severe opportunistic infection. Once in postnatal care for her own health, ART eligibility was assumed to be assessed through both CD4 testing and clinical evaluation. For women identified as ART-eligible, both ART and trimethoprim/sulfamethoxazole prophylaxis were initiated [13], [35]. For women identified as not yet ART-eligible in postnatal HIV care, medications were administered depending on the modeled breastfeeding prophylaxis regimen: no medications were dispensed for the “no antenatal ARVs” and “sdNVP” regimens; infant nevirapine syrup was dispensed for the “Option A” regimen; and maternal ART was dispensed for the “Option B” and “Option B+” regimens. Specific components of 3-drug ART regimens were simulated to reflect 2009 Zimbabwean guidelines and common current practice in Zimbabwe (Text S1 and Table 1) [35]. ART monitoring and switching strategies are also detailed in the Appendix (Text S1).

#### Loss to follow-up

During modeled ANC, women could be lost to follow-up (LTFU) at any stage between first presentation (booking) and delivery; if LTFU, no antenatal ARVs were received, but the opportunity to access HIV testing and sdNVP in labor remained. Women could also be LTFU between delivery and six weeks postpartum [36], or after linkage to postnatal HIV care [37], [38]. In the absence of specific maternal or pediatric data regarding monthly risks of LTFU and cessation of prophylactic ARVs during breastfeeding, the impact of such events was incorporated in sensitivity analyses via the highest published postnatal transmission estimates for Options A, B and B+. For HIV-infected infants, the impacts of loss to follow-up after established pediatric HIV care were included in cohort-based pediatric survival estimates.

### Model input parameters

#### Maternal cohort characteristics and natural history

Baseline maternal characteristics reflected cohorts of pregnant women in Zimbabwe (Table 1 and Text S1). At first ANC visit, mean age was 24.0 [27] and HIV prevalence was 16% [39]; HIV incidence during pregnancy and breastfeeding was 0.96%/year [39]. Among HIV-infected women, mean CD4 was 275 cells/µL if ART-eligible [21], 550 cells/µL if not ART-eligible [21], and 664 cells/µL if incidently infected during pregnancy [40]. Because detailed clinical data to inform HIV disease progression in the absence of ART were not available from Zimbabwe, these natural history model inputs to the CEPAC model were derived from a clinical cohort in South Africa (Text S1) [41]. For women remaining HIV-negative, life expectancy was projected using UNAIDS cause-deleted mortality rates [42].

#### Maternal access to care and ART

Modeled rates of access to ANC, HIV and CD4 tests in ANC, ARV drugs for PMTCT, and postnatal care reflected Zimbabwean national estimates whenever available (Table 1). In the sdNVP strategy, women were modeled to undergo clinical ART-eligibility assessment, but not CD4 testing, and to initiate lifelong ART if WHO stage 3–4 disease was identified [11]. The sensitivity of clinical assessment for ART eligibility was 36% [43]. In the base case analysis, to isolate the benefits of each regimen, ARV medications were assumed to be available for, accepted by, and adhered to by all women diagnosed as HIV-infected in ANC, and CD4 testing (but not result return) was assumed for all women under Option A. In addition, sdNVP was modeled to be provided to 80% of women newly HIV-diagnosed in labor [23], [39].

The base-case rate of linkage to postnatal maternal HIV care reflected the median of published values; in sensitivity analyses, linkage rates ranged from 51–100%, depending on antenatal care and PMTCT regimen received [36], [44]–[48]. For women in HIV care and receiving ART, the 24-week “efficacy” of first-line, NNRTI-based maternal ART in suppressing HIV RNA to <400 copies/ml was 85% for women with previous sdNVP exposure [49] and 90% (assumed difference of 5%) for women without exposure to sdNVP [50]. In the six months following ART interruption at weaning (Option B), CD4 count was modeled to decline by 139 cells [30], [31]. Other modeled ART effects are described in Table 1.

#### Mother-to-child transmission risks

Mother-to-child HIV transmission risks were derived from randomized clinical trials in several African settings (Table 2) [1], [4], [5], [8]–[10], [15], [17], [21], [51]–[61]. MTCT risks during the intrauterine and intrapartum period (by 4–6 weeks of age) and postpartum period (6 weeks-18 months) were stratified by maternal HIV stage and by PMTCT regimen received. Postpartum transmission risks were additionally stratified by whether breastfeeding during the first six months of life was exclusive (EBF) or mixed (MBF, including any non-breastmilk liquid or solid) [62].

**Table 2:** Selected model input parameters for a simulation model of strategies to prevent mother-to-child transmission of HIV in Zimbabwe: Pediatric data.

II. Mother-to-child transmission risks				
	Base Case Value (Range for sensitivity analysis)			
Maternal HIV status	PMTCT regimen received			
Intrauterine/intrapartum period (one-time risks)				
	No antenatal ARVs	sdNVP*	scZDV	ART
Mother ART eligible at conception	0.273 [52], [83](0.199–0.322) [54], [83], [84]	0.176 [21], [85](0.082–0.264) [15], [17], [86]	0.136 [5](0.091–0.157) [87], [88]	0.033 [89](0.011–0.041) [10], [58]
Mother not ART-eligible at conception	0.175 [52], [83](0.127–0.206) [54], [83], [84]	0.073 [21], [85](0.033–0.109) [15], [17], [86]	0.036 [5](0.024–0.041) [8], [88]	0.01 [10](0.004–0.028) [4], [10]
Mother with incident infection during pregnancy	0.30(assumption)	0.20 (assumption)	0.16(assumption)	0.033(assumed = eligible)

Postnatal period (monthly risks, among infants HIV-uninfected at 4–6 weeks of age)			
	No postnatal ARVs	Maternal ART	Extended infant NVP
Mother ART eligible at conception	0.00758 (EBF) [21], [85];0.01278 (MBF) [21], [85];(0.00476–0.02336) [53], [84]	0.00333[87](0–0.00534) [4], [8], [10]	n/a
Mother not ART-eligible at conception	0.00238 (EBF) [85]; 0.00401 (MBF) [21](0.00149–0.00732) [53], [60]	0.00189 (average: [10], [46], [56], [57])(0–0.00534) [9], [10]	0.00221 [9], [61](0.00120–0.00311) [9], [61]
Mother with incident infection during pregnancy	0.00758 (EBF) [85];0.01278 (MBF) [21], [85];(0.00476–0.02336) [53], [84]	n/a	n/a

III. Pediatric inputs			
Variable	Base Case Value	Range for sensitivity analyses	Data sources
Postnatal HIV Care			
Probability of linking to pediatric HIV diagnosis, care, and ART	36%	0–100%	WHO [64]

Mortality risks	One-year risk (%)	Two-year cumulative risk (%)	
HIV-unexposed children	5.4	5.9	[42]
HIV-exposed, uninfected children	7.4	9.2	[63]
HIV-infected children, no ART			
Intrauterine/intrapartum infection	51.0	65.0	[6]
Postpartum infection	24.0	38.0	[6]
HIV-infected children, on ART	9.5	12.0	[65], [90]
Relative increase in infant mortality if maternal death occurs before 18 m	2-fold increase (range: 1–6-fold increase)		[33], [34]

SD: Standard deviation; MOHCW: Zimbabwe Ministry of Health and Child Welfare; MACS: Multicenter AIDS Cohort Study; CTAC: Cape Town AIDS Cohort; ANC: antenatal care; ART: 3-drug, combination antiretroviral therapy; (sd)NVP: (single-dose) nevirapine; scZDV: short-course zidovudine; WHO: World Health Organization; ZDV: zidovudine; 3TC: lamivudine; d4T: stavudine; LPV/r: lopinavir/ritonavir; TDF: tenofovir; FTC: emtricabine; EBF/MBF: exclusive/mixed breastfeeding.

*“Without sdNVP exposure” refers to situations in which sdNVP was not received, or was received in combination with short-course zidovudine or other antenatal/intrapartum medications which may decrease risk of NVP-associated NNRTI-resistant HIV [19].

#### Pediatric outcomes

Mortality estimates for HIV-unexposed children were derived from UNAIDS HIV-deleted mortality estimates [42] and mortality rates for exposed-uninfected infants were from the ZVITAMBO study in Zimbabwe [63] (Table 2). Mortality rates for untreated HIV-infected children, stratified by timing of HIV infection, were derived from a patient-level analysis of 1,930 children from 12 African PMTCT trials, after removing non-HIV-related causes of death [6]. ART was assumed available for 36% of HIV-infected children [64]. Mortality rates for children treated with ART were from a systematic review of pediatric ART in Africa [65] and a pooled patient-level analysis from 16 African cohorts [66]. For infants of any HIV infection or exposure status, maternal death was assumed to increase mortality 2-fold [33].

### Model validation and sensitivity analyses

Model results were validated by comparison to published values (Text S1). Sensitivity analyses, described in detail in the Appendix (Text S1), examined the impact of variations in key maternal clinical and demographic characteristics, highest and lowest published MTCT risks for each regimen, access to ANC and HIV testing in ANC, linkage to postnatal HIV care, ART efficacy and survival following sdNVP exposure, CD4 cell decline following ART interruption, and pediatric and maternal non-HIV-related mortality rates. Parameters leading to substantial changes in model results were identified; a substantial change was defined as 1) a change in the relative order of the outcomes of the PMTCT regimens, or 2) a >10% relative change in the difference between projected outcomes for each ARV regimen (example provided in Text S1). In addition, we projected the impact of each PMTCT regimen on infant infection risk for an annual cohort of women becoming pregnant in Zimbabwe, including both HIV-infected and HIV-uninfected women at conception (Text S1).

### Role of the funders and ethics approval

The funders had no role in study design, data collection and analysis, decision to publish, or preparation of the manuscript. This analysis was deemed “not human subjects research” by the Partners Healthcare IRB, and was approved as an exempt analysis.

## Results

### Base-case analyses (fixed levels of ARV uptake for PMTCT)

#### Pediatric outcomes

Among women HIV-infected at first ANC visit, the risks of infant infection at 4–6 weeks of age were projected to be 20.1% with no **antenatal** ARV prophylaxis, 10.8% with the 2002–2009 sdNVP-based national program, 7.2% with Option A, and 5.4% with Option B and B+ (Table 3, top). At 18 months of age, cumulative risk of infant HIV infection ranged from 25.8% (no antenatal ARVs) to 10.9% (Option B/B+). Two-year pediatric survival similarly ranged from 78.4% (no antenatal ARVs) to 84.9% (Option B/B+).

**Table 3:** Outcomes of strategies to prevent mother-to-child HIV transmission in Zimbabwe: Base-case model results.

	Model results		
Pediatric outcomes (Infants born to women who are HIV-infected at conception)			
	Risk of HIV infection at 4–6 weeks of age (%)	Risk of HIV infection at 18 months of age (%)	2-yearsurvival (%)
No **antenatal** ARVs*	20.1	25.8	78.4
Single-dose NVP	10.8	17.2	81.9
Option A	7.2	12.8	83.5
Option B	5.4	10.9	84.9
Option B+	5.4	10.9	84.9

Maternal outcomes after delivery (women who are HIV-infected at conception)			
	2-yearsurvival (%)	5-yearsurvival (%)	Life expectancy (years)
No **antenatal** ARVs*	92.4	79.8	**14.0**
Single-dose NVP	92.4	79.8	**13.8**
Option A	92.5	79.9	**14.0**
Option B	93.7	80.0	**13.9**
Option B+	94.0	82.2	**14.5**

* “No antenatal ARVs” refers to the receipt of no medications for HIV therapy or for prevention of mother-to-child transmission during the antenatal period. Women who link to postnatal HIV care are assumed to start ART after delivery if CD4 cell count is ≤350/µL or following development of a severe opportunistic infection.

#### Maternal outcomes

Postpartum survival among HIV-infected women was projected to range from 92.4% (no antenatal ARVs, sdNVP) to 94.0% (Option B+) at two years, and 79.8% (no antenatal ARVs, sdNVP) to 82.2% (Option B+) at five years after delivery (Table 3, bottom). The impacts of sdNVP-associated resistance and ART interruption manifested primarily after five years postpartum: projected life expectancy was slightly lower following sdNVP (13.9 years) or Option B (13.9 years) than following receipt of no antenatal ARVs or Option A (14.0 years). Projected maternal life expectancy was greatest (14.4 years) with lifelong universal ART (Option B+).

### Sensitivity analyses

**Figure 2:**
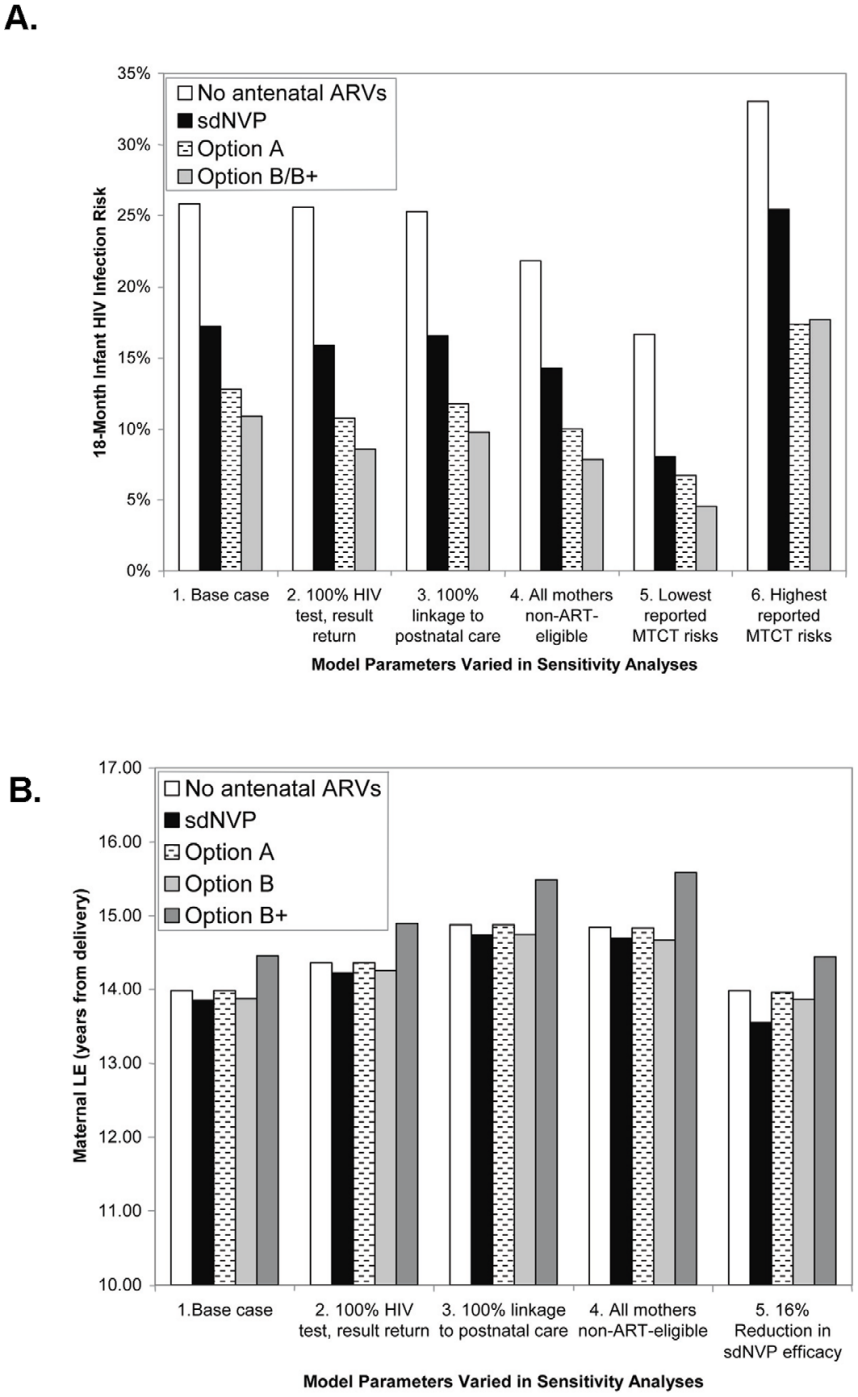
Key sensitivity analyses, identifying selected parameters producing substantial changes in model results. As detailed in the Methods section and Appendix (Text S1), a substantial change in results was defined as: 1) a change in the relative order of the outcomes of the PMTCT regimens, or 2) a >10% relative change in the difference between projected outcomes for each regimen. Panel 2a depicts parameters influencing 18-month mother-to-child HIV transmission risk, and Panel 2b depicts parameters influencing maternal life expectancy from delivery; these outcomes are shown on the vertical axes. Along the horizontal axes, each group of vertical bars represents a single scenario (numbered 1–6 in 2a and 1–5 in 2b), and each vertical bar represents a PMTCT regimen, as indicated.

**Figure 3:**
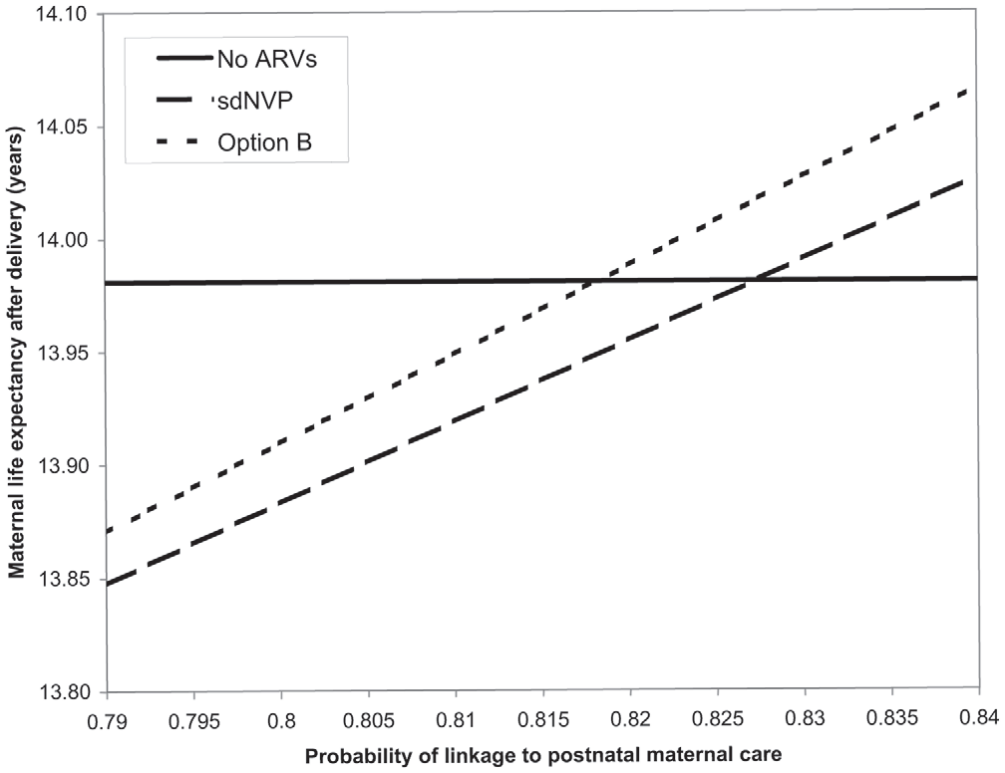
Sensitivity analysis demonstrating the impact on maternal life expectancy of rates of linkage to postnatal HIV care. The vertical axis represents maternal life expectancy, in years from delivery. The horizontal axis depicts the probability of linkage to postnatal HIV care for women who receive the PMTCT regimens shown. This probability of linkage to care is varied from 79% (the base case value) to 85%. In the base case, maternal life expectancy following the sdNVP and Option B regimens is lower than if no antenatal ARVs were received for PMTCT (triangles). This occurs as a result of modeled negative impacts of sdNVP-associated resistance (sdNVP regimen) and ART interruption (Option B regimen). When the probability of linkage to care following Option B is ≥81.8%, as indicated by the open arrow (2.8% more than if no antenatal ARVs are received), the negative impact of ART interruption is overcome. Similarly, when the probability of linkage to care following sdNVP is ≥82.8%, as indicated by the solid arrow (3.8% more than if no ARVs are received), the negative impact of sdNVP-associated resistance is overcome.

Sensitivity analyses were performed on all key model input parameters and assumptions (Figures 2 and 3, Text S1). The model input parameters with the greatest influence on pediatric and maternal outcomes are shown in Figure 2 (Figure 2a: 18-month infant infection risk; Figure 2b: maternal life expectancy). Improvements in HIV testing during pregnancy (2a and 2b: scenario 2), linkage to postnatal care (2a and 2b: scenario 3), and reduction in the proportion of women with CD4<350 (2a and 2b: scenario 4) as might result through universal HIV screening acceptance and early ART initiation prior to pregnancy) led to the greatest improvements in both maternal and pediatric outcomes. Lowest published MTCT risks for each regimen (2a: scenario 5) did not change the relative order of the infant outcomes of the PMTCT regimens, but markedly reduced overall transmission risks; lowest projected 18-month infection risks ranged from 8.1% (sdNVP) to 4.5% (Option B/B+).

In the base case (Table 3), maternal life expectancy was slightly shorter following receipt of sdNVP (13.8 years) than if no antenatal ARVs were received for PMTCT (14.0 years). The maternal survival and life expectancy differences between no antenatal ARVs and sdNVP also depended on the degree to which sdNVP exposure was assumed to reduce subsequent 1^st^-line ART efficacy; at 0% difference, the regimens led to equal maternal outcomes (Appendix); at 16% difference, maternal outcomes associated with each strategy differed substantially (Figure 2b, scenario 5). Base-case maternal life expectancy was also shorter following receipt of Option B than if no antenatal ARVs were received (Table 3). This result did not change when a wide range of reported rates of CD4 decline after ART interruption were incorporated (Appendix) [30]–[32].

Figure 3 depicts the impact of linkage to postnatal HIV care on maternal life expectancy. In the base case, rates of linkage to postnatal care were assumed to be equal following receipt of any PMTCT regimen. As noted above, resulting maternal life expectancies were slightly lower following the sdNVP and Option B regimens than if no antenatal ARVs were received for PMTCT. However, if the receipt of sdNVP or Option B improved rates of linkage to postnatal care even slightly, compared to ANC without receipt of antenatal ARVs for PMTCT (2.8% increase in linkage for Option B, 3.8% increase for sdNVP), the negative impact of these regimens on life expectancy was overcome.

All other parameters varied in sensitivity analyses are detailed in the Appendix (Text S1). Notably, policy conclusions were insensitive to variations in maternal age, risk of maternal mortality due to pregnancy, probability of live infant birth, delivery location, impact of sdNVP exposure on short-term infant outcomes, and relative risk of infant mortality following maternal death.

## Discussion

We linked two validated computer simulation models to project clinical outcomes of improved PMTCT regimens in Zimbabwe. These analyses simultaneously evaluate pediatric and maternal outcomes, a novel approach for model-based investigations of HIV care [67], [68], and are likely applicable to other sub-Saharan Africa settings where prolonged breastfeeding is common. Altogether, pediatric outcomes, including HIV infection risk and survival, would be markedly improved by replacing sdNVP-based programs with 2010 WHO guideline-concordant regimens at fixed levels of ARV uptake for PMTCT. However, it is worth noting that the largest improvements in both pediatric and maternal survival may result from a PMTCT program that offers lifelong ART to all pregnant, HIV-infected women regardless of CD4 count or disease stage (Option B+), as is currently being investigated [28] and already being considered for implementation in Malawi [12].

The choice of ARV regimen for PMTCT may have important effects on maternal health, important not only in its own right, but also for its impact on pediatric survival [33], [34]. First, this analysis underscores the potential long-term impact of sdNVP exposure. The modeled impacts of ARV regimens that are received only briefly during pregnancy or breastfeeding are small in comparison to the impact of lifelong ART for women who link to postnatal HIV care. Nonetheless, the sdNVP-associated life expectancy reduction of **0.1** years is comparable in magnitude to the modeled benefit of trimethoprim/sulfamethoxazole prophylaxis [25]. This life expectancy reduction results from reduced virologic suppression on NNRTI-based ART due to sdNVP-associated NNRTI-resistant virus. The difference in suppression among women with and without sdNVP exposure determines the degree to which sdNVP-based programs create a tradeoff between improved pediatric outcomes and reduced maternal life expectancy. The impact of sdNVP exposure may be negligible if sdNVP is received >12–24 months prior to ART initiation [49], [69]–[71]; in such cases, scale-up of sdNVP-based PMTCT services will improve both maternal and pediatric outcomes. However, absolute reductions in 24-week virologic suppression rates of 16–42% and 12–14% have been reported for women initiating ART <6 months and 6–12 months after sdNVP exposure, respectively [18], [49], [69] (base-case value for this analysis: 5%). Because protease inhibitor-based first-line ART is not widely available in many settings, the current analysis lends strong support to efforts to expand non-sdNVP-based PMTCT programs to improve both maternal and child health.

In addition to the impact of sdNVP exposure, the ART initiation and discontinuation strategies required by Option B may also impact maternal health. Because ART is recommended for all pregnant, HIV-infected in women in Option B, there may be a health benefit from initiating ART at higher CD4 counts than would prompt therapy in non-pregnant patients. However, it is not yet known whether the adverse effects of ART discontinuation after weaning will outweigh these benefits. When ART is interrupted in men and non-postpartum women, marked increases in viral load, inflammatory markers, and risk for both AIDS- and non-AIDS-related events have been reported [30], [72]. The current analysis models rapid CD4 declines, ranging from 75–139 cells/µL, in the 6 months following ART interruption at weaning in Option B [30], [32], [72]; this results in a decrease in maternal life expectancy of 0.6 years for women who interrupt ART at weaning (then resume when later required for their own health), compared to women who continue ART. In the absence of randomized data from postpartum women, we do not specifically simulate additional increased rates of non-AIDS-related events due to interruption; if additional non-AIDS-related risks exist, the negative impact of Option B will be greater than shown here. Data on AIDS- and non-AIDS-related events among postpartum women continuing or interrupting ART are anticipated in the next several years [28], and will more accurately inform the risks and benefits of Option B versus Option B+.

These analyses also highlight that linkage to postnatal maternal HIV care, and thus initiation of ART when needed, is the step in the PMTCT “cascade” that most dramatically influences maternal life expectancy. The impact of improved linkage to care on maternal life expectancy is greater than that of improved access to ANC or HIV testing (holding linkage rates constant for those in care and diagnosed), or any specific PMTCT regimen during pregnancy. Because published rates of linkage to postnatal care range widely and are rarely stratified by PMTCT regimen received [46], [47], model-based analyses can highlight areas for future research. For example, if women who receive sdNVP- or Option B-based PMTCT interventions are even slightly more likely to register in postnatal HIV care than are women who receive ANC but no medications for PMTCT (3–4% absolute increases in linkage rates), the life expectancy benefits of this improvement in linkage outweigh the negative effects of sdNVP-associated resistance and ART interruption. However, if receipt of these regimens does not improve linkage to postnatal care compared to ANC services alone, there may be an important tradeoff between long-term maternal and pediatric health with expansion of sdNVP- or Option B- based programs.

Although linkage to postnatal care was the access-to-care parameter with the greatest individual influence on maternal life expectancy, improved uptake at each step in a “cascade” of PMTCT care (including access to ANC, HIV testing and result receipt, and availability and acceptance of ARVs [73]; Figure 1) confers substantial benefits for both infant and maternal health. In fact, improvements in two dimensions are critical: both replacement of sdNVP with more effective regimens and improved uptake of any given PMTCT regimen. While the current analysis addresses the first dimension in greatest detail, further analyses examining the clinical impacts, costs, and cost-effectiveness of interventions to improve uptake are also needed.

This analysis has two main limitations. First, when detailed clinical data to inform modeled maternal and pediatric HIV disease progression were not available from Zimbabwe, data from surrounding southern African countries were used instead. Base-case clinical risks for HIV-infected mothers, such as risks of opportunistic infections and tuberculosis, were derived from South Africa, and might reasonably be expected to be similar in neighboring Zimbabwe. Similarly, risks of MTCT, after stratification by maternal CD4, PMTCT regimen received, and breastfeeding duration, might also be anticipated to be similar across Southern African settings. The impacts of non-Zimbabwean data were tested in extensive sensitivity analyses, as detailed in the Appendix (Text S1), and had little impact on model results. Second, life expectancy projections are subject to uncertainty about events occurring over long time horizons, as healthcare systems and HIV therapy may change substantially in the distant future. Because of the uncertainty inherent in long-term projections, we also report similar findings based on the short-term outcomes of MTCT risks, 2-year pediatric survival, and 2-year and 5-year maternal survival.

Model-projected clinical outcomes are not intended to be interpreted in isolation, but rather as one component of a decision-making framework that incorporates many factors, including feasibility, affordability, and equity. Implementation of more effective PMTCT regimens will be resource-intensive; costs will include not only drug costs, but also infrastructure, personnel, and training costs for clinics and laboratories. However, the 2010-recommended PMTCT regimens may also save money in the future by averting not only costly care for pediatric HIV infection [74], but also the costly morbidity and mortality associated with delayed maternal ART initiation [26], [75] or sdNVP-associated resistance [29]. Model-based analyses of pediatric outcomes have suggested that ART for PMTCT is very cost-effective in many settings, compared to sdNVP or to no ARVs [76]–[78]. Additional budgetary impact and cost-effectiveness analyses, comparing Option A to Option B and incorporating short-term and long-term benefits and costs for both mothers and infants, will comprise an important area of future research. In the interim, model-based analyses can assist policymakers to understand the clinical tradeoffs anticipated to result from the replacement of sdNVP with more effective PMTCT regimens.

### Conclusions

Replacing sdNVP with currently WHO-recommended PMTCT regimens at a fixed level of ARV uptake for PMTCT will improve both maternal and pediatric health outcomes in Zimbabwe. The best currently available data suggest that continued dependence on sdNVP-based programs would impact positively on pediatric health, but may worsen long-term maternal outcomes, unless also accompanied by expanded access to postnatal HIV care and ART for eligible women. To avoid an untenable tradeoff between maternal and child health, additional resources are needed to implement WHO guideline-concordant PMTCT programs (Options A, B, or possibly B+) in resource-constrained settings, including Zimbabwe.

## Supporting Information

Text S1 The Appendix reports additional details of technical model structure, input data parameters, and model results, including extensive model validation and sensitivity analyses.
(DOC)
