# Supplementary material for: WHO 2010 Guidelines for Prevention of Mother-to-Child HIV Transmission in Zimbabwe: Modeling Clinical Outcomes in Infants and Mothers
Source: PLoS One. 2011 Jun 2;6(6):e20224. doi: 10.1371/journal.pone.0020224 (PMC3107213; doi:10.1371/journal.pone.0020224)
Supplement: Text S1 — The Appendix reports additional details of technical model structure, input data parameters, and model results, including extensive model validation and sensitivity analyses. (DOC) [file pone.0020224.s001.doc]

**WHO 2010 guidelines for prevention of mother-to-child HIV transmission in Zimbabwe: Modeling clinical outcomes in infants and mothers**

**Supplemental Appendix**

Andrea L. Ciaranello, MD, MPH, *et al.*

**INTRODUCTION**

# This appendix is included to provide methodologic details to supplement the description of the methods in the manuscript text, as well as additional model output and results.

**METHODS**

*Population*

The manuscript reports model results for a population of women who are chronically HIV-infected at first ANC visit. We also examined a cohort of women representative of the entire Zimbabwean population, with mean age 24.0 years, HIV prevalence 16%, and HIV incidence of 0.96%/year .

*PMTCT strategies evaluated*

Details of the five PMTCT strategies evaluated are described in Supplemental Table 1.

*MTCT model structure*

The previously published MTCT model was expanded to include all steps in the PTMCT "cascade" (Manuscript Figure 1 and Supplemental Figure 1), including presentation to ANC; offer and acceptance of HIV testing; receipt of HIV test results; clinical assessment for ART eligibility; CD4 testing and receipt of results; offer of, acceptance of, and adherence to ARVs for PMTCT; linkage to postnatal care and ART for mothers; and linkage to HIV care and ART for infants.

***CEPAC model structure***

In addition to the technical details provided in this Appendix, further information regarding the Cost-effectiveness of Preventing AIDS Complications (CEPAC) model have been included in several previous publications and the accompanying Technical Appendices . In brief, the adult CEPAC model is a first-order Monte Carlo simulation model of HIV infection. HIV-infected women are simulated individually from model entry through death. Disease progression is characterized by a series of monthly transitions between health states, including acute opportunistic infections and death. The model records all clinical events during each patient's lifetime, and produces aggregate estimates of life expectancy (mean) and survival probabilities for the simulated cohort. The infant CEPAC model is a simplified first-order Monte Carlo simulation model, incorporating the risk of infant HIV infection and infant all-cause mortality during the first two years of life. Details of both the infant and adult models are shown in Supplemental Figure 2.

*ART in the adult CEPAC model*

In the adult CEPAC model, virologic failure on ART may occur either “early” (<24 weeks) or “late” (>24 weeks) after ART initiation. The resulting rise in HIV RNA reflects “true” virologic failure for simulated patients, and is distinct from the ability to detect “observed” failure through HIV RNA monitoring. After ART interruption (Option B), CD4 count declines rapidly (mean, 139 cell decline) over the first 6 months . Thereafter, CD4 count declines at the “natural history” rate (as before ART was initiated) .

In this analysis, reflecting 2009 Zimbabwean guidelines, women in HIV care underwent outpatient clinical evaluation every two months and CD4 monitoring biannually; HIV RNA monitoring was not available . ART regimens were simulated to reflect 2009 Zimbabwean guidelines and common current practice in Zimbabwe (Manuscript

Table 1): if initiated during pregnancy, the first-line ART regimen included nevirapine (NVP) with lamivudine and zidovudine; if initiated postpartum, first-line ART included NVP, lamivudine, and stavudine . The second ART regimen, if needed, included lopinavir/ritonavir (LPV/r) with tenofovir/emtricitabine (TDF/FTC) . Following WHO recommendations, modeled patients were switched from the first to second antiretroviral regimen after observed clinical or immunologic failure, defined as at least one severe opportunistic infection (defined in Supplemental Table 2), a ≥50% decrease from the peak on-ART CD4 count, or an absolute CD4 count <100 cells/µL . After failure of 2^nd^-line ART, defined as three severe opportunistic infections or a ≥90% decrease from peak on-ART CD4 count, this regimen was continued unless severe toxicity

occurred .

*Model input parameters*

Model input parameters are shown in Manuscript Tables 1 and 2 and Supplemental Table 2. MTCT risk parameters in breastfeeding populations were determined using the following approach:

1. Where possible, pooled estimates from formal meta-analyses were used .

2. Where pooled estimates were not available, estimates were prioritized as follows:

a. If studies reported directly on the variable of interest with appropriate CD4 stratification, these were used for the base case .

b. If several studies reported estimates using CD4 cutoffs other than 350/µL, we chose the midpoint of available estimates for the base case value, and the upper and lower ranges for the highest and lowest MTCT risk sensitivity analyses, respectively.

c. Where data were not available stratified by maternal CD4 greater/less than 350, we attempted to find data to calculate a relative risk of transmission among maternal CD4 greater/less than 350/µL from another source, then applied this relative risk to the study selected. For ART in pregnancy, we were not able to find studies reporting MTCT stratified by maternal CD4 greater/less than 350/µL, or any papers allowing us to calculate a relative risk of transmission among maternal CD4 greater/less than 350/µL. We therefore used the midpoint of the published range for the base case, highest values for risk among women with CD4 ≤350/µL, and lowest values for risk among women with CD4 >350/µL.

***Determination of simulated cohort size***

A cohort size of ten million women was selected in order to produce stable estimates of outcomes. The CEPAC models are stochastic models (risks of clinical events are drawn individually for each simulated patient from a specified distribution of numerical values). As a result, a model simulation with a small number of patients will generate a large amount of variation in model outcomes: when the same simulation is repeated several times using identical input parameters, model outcomes will differ substantially between each simulation. However, repeated model simulations using large numbers of simulated patients will lead to convergence of results. Cohort sizes of one million to ten million patients are generally sufficient for life expectancy estimates over repeated simulations to converge to values that differ by less than 0.01 life year. Stable, model-generated results from these large simulations can then be applied to specific, smaller populations. For example, risks of infant infection (as percentages) can be multiplied by an annual number of pregnancies to estimate the number of HIV-infected infants in a one-year period. An example of this calculation is shown in Supplemental Table 5, using the estimated number of total pregnancies in Zimbabwe each year (392,460 in 2009, including both HIV-infected and HIV-uninfected women) .

*Model validation*

Model outcomes of pediatric HIV infection at birth and 18 months were validated internally by comparison to the data from the PMTCT trials that served as input parameters, and externally by comparison to data from other African PMTCT trials . Pediatric survival at 2 years of age was validated internally by comparison to data from Zimbabwe and externally by comparison to data from other African countries . Adult survival estimates generated by the CEPAC model have been validated previously .

*Sensitivity analyses*

Sensitivity analyses examined the impact of variations in maternal age and CD4 count; maternal perinatal mortality; and rates of live birth (data ranges for sensitivity analyses are shown in Manuscript Tables 1 and 2). Lowest- and highest- MTCT risk scenarios were simulated, encompassing the range of reported MTCT risks for each ARV regimen. Non-HIV-related mortality rates for HIV-infected and HIV-uninfected women and children were also varied through wide ranges , as was the impact of maternal mortality on infant survival . Because of growing concern about reduced efficacy of nevirapine-based ART following sdNVP exposure , the impact of sdNVP-associated resistance on maternal 1^st^-line ART efficacy and infant survival was varied. To address uncertainty in the rate of CD4 cell decline after ART interruption, analyses were repeated using the slower CD4 cell decline observed in the Kesho Bora study . Finally, to evaluate the effects of improved access to care, we varied rates of access to ANC, of HIV testing and result receipt in ANC, and of linkage to postnatal maternal and infant HIV care and ART.

***Definition of model parameters producing "substantial" changes in model results.*** In order to highlight parameters with the most important effects on model results, we used a standardized, two-component definition of a "substantial" changes in model results. First, results were deemed to have changed substantially if the relative order of the outcomes of the PMTCT regimens changed from the base case (for example, scenarios in which maternal life expectancy following sdNVP was no longer shorter than following no antenatal ARVs). Second, if the order of regimens was unchanged, a substantial change was said to have occurred if there was a >10% relative increase or decrease in the degree to which one regimen was superior to another, compared to the base case. This criterion avoids identification as influential a parameter that increases or decreases projected results equally for all regimens, but does not change the relative magnitude of the differences between the outcomes for each regimen. An example of the application of this definition is provided in Supplemental Table 7.

**RESULTS**

*Model validation (Supplemental Table 3)*

Modeled risks for infant and childhood mortality closely match published Zimbabwean and pooled African data . For each PMTCT regimen evaluated, MTCT model results fall within the ranges of published transmission risks at birth and 18 months; where MTCT data have not been reported to 18 months of age, 18-month model results exceed published risks by an anticipated order of magnitude (Supplemental Table 3) .

*Intermediate (CEPAC adult) model results*

CEPAC adult model outputs of 18-month mortality and life expectancy for HIV-infected and –uninfected mothers are shown in Supplemental Table 4. These CEPAC model outputs comprised inputs to the MTCT model.

*General Zimbabwean population results*

When projected for the general Zimbabwean population, including chronically HIV-infected women, incidently HIV-infected women, and HIV-uninfected women, improved PMTCT regimens had a noticeable impact on maternal and pediatric outcomes (Supplemental Table 5). Risk of HIV infection among newborns ranged from 1.1% (Option B and B+) to 3.4% (no antenatal ARVs) and among 18 month-old infants from 2.1% (Option B and B+) to 4.5% (no antenatal ARVs). At this general population level, more intensive PMTCT regimens led to small but detectable differences maternal life expectancy after delivery (43.1-43.2 years); these maternal life expectancy gains are comparable to the population-level impacts of common health-related interventions in developed countries . However, more intensive PMTCT regimens led only to small differences in short-term outcomes (pediatric 2-year survival (89.0-90.0%), maternal 2-year survival (97.7-98.0%), and maternal 5-year survival (94.8-95.2%)). These analyses highlight that, with a 16% HIV prevalence in Zimbabwe, more effective PMTCT regimens are anticipated to lead to HIV transmission and survival benefits detectable even at the general population level.

*Sensitivity analyses*

Results of sensitivity analyses are shown in Manuscript Figures 2 and 3, and Supplemental Table 6. Section A of Supplemental Table 5 reiterates the base case results, for comparison; Sections B and C include model parameters with substantial influence on results of the analysis; and Section D lists the model parameters that did not change policy conclusions.

**Supplemental Figure 1. Schematic representation of the MTCT model.**

**LEGEND:** The MTCT model is a decision tree, coded in TreeAge Pro software. Pregnant women enter the model at conception. The five modeled PMTCT strategies are shown at the decision node, indicated by a square. Circles indicate chance nodes, at which events occur based on probabilities derived from published literature. Triangles indicate terminal nodes, representing the clinical outcome of any single pathway through the model. Brackets reflect that the subsequent events emerging to the right of the bracket may follow any of the prior chance nodes included to the left of the bracket. At each chance node, the probabilities of all subsequent modeled events may depend on the PMTCT strategy being simulated and on the prior events leading to that node.

For each modeled PMTCT strategy, the series of events shown in the Figure may occur. For example, women may be HIV-infected or HIV-uninfected at conception (this probability is the HIV prevalence in antenatal care). If HIV-infected, they may be ART-eligible (CD4≤350/µL or WHO Stage 3-4 disease) or non-ART-eligible; ART-eligibility may be identified by CD4 testing, identified by clinical evaluation, or not identified. All women may access ANC, undergo HIV testing in ANC, and receive HIV test results, or may fail to access these steps in the cascade.

If identified as HIV-infected (correctly or incorrectly), women may be offered ARVs for PMTCT according to the PMTCT strategy being simulated, as well as ART if identified as ART-eligible (not shown). Probabilities for surviving pregnancy depend on receipt of ART; if maternal death occurs, infant death also occurs. Women who survive pregnancy may deliver at a healthcare facility or at home; if they deliver in a healthcare facility, they may access HIV testing (if previous status was unknown or negative), and if identified as HIV-infected at that time, may receive sdNVP in labor. All women surviving pregnancy then experience probabilities of live birth and HIV infection in the infant, depending on PMTCT regimen received. Finally, women may link or fail to link to postnatal HIV-related care for themselves.

At the end (far right) of any given path through the model, there are two sets of outcomes: infant outcomes and maternal outcomes. Infant outcomes include HIV infection status (infected or uninfected at birth, shown), risk of postnatal HIV infection if uninfected at birth, and 2-year survival. Maternal outcomes include 2-year survival, 5-year survival, and life expectancy. These outcomes are derived from the CEPAC infant and adult models, through specific simulations of each possible scenario shown at the end of each pathway in the MTCT model. As an example of maternal outcomes from the CEPAC adult model (Supplemental Figure 2a and Supplemental Table 4), an ART-eligible mother who links to postnatal care (and thus begins ART) will face monthly risks of disease progression or death based on treated HIV infection. As an example of infant outcomes from the CEPAC infant model, an HIV-uninfected infant with an ART-eligible mother who is in postnatal care (and thus on ART) would face monthly risks of HIV infection based on receipt of maternal ART during breastfeeding. These CEPAC model outputs are then used as “payoffs” (outcomes) in the MTCT model, according to conventional methods for evaluation of a decision tree. The average value assigned to any modeled PMTCT strategy in the MTCT model is essentially a weighted average of the value of these “payoffs” at the end of each pathway (weighted by the probabilities of reaching each possible path endpoint).

**Supplemental Figure 2. Schematic representations of the CEPAC Adult and Infant Models**

**LEGEND**. Supplemental Figure 2 shows schematic representations of the adult and infant CEPAC model structures.

Women enter the adult model (Figure 2a) after delivery, either during acute infection (incident infection during the last trimester of pregnancy) or chronic HIV infection. They then face monthly risks of clinic events including opportunistic infections, medication toxicities, and death; these risks are stratified by the parameters listed in the figure.

Infants enter the infant model (Figure 2b) after birth, either as HIV-unexposed infants (mothers are uninfected), HIV-exposed but uninfected infants, or infants infected during the intrauterine/intrapartum period. Unexposed infants become exposed if maternal incident HIV infection occurs during breastfeeding; exposed-uninfected infants become postnatally infected if breastfeeding transmission occurs. From any infection state, infants face a risk of all-cause mortality. Monthly risks of infant HIV infection and infant mortality are stratified by the parameters listed in the figure.

**Supplemental Table 1. Regimens evaluated in a simulation model of strategies to** prevent mother-to-child transmission of HIV in Zimbabwe

| **PMTCT**  **Strategy** | **Antenatal**  **(maternal)** | **Intrapartum**  **(maternal)** | **Postpartum**  **(maternal)** | **Neonatal**  **(breastfeeding infant)** |
| --- | --- | --- | --- | --- |
| **No antenatal ARVs** | None | None | None | None |
| **sdNVP (2002-2009 National Program)** | *CD4≤350/µL or WHO Stage 3-4:* ZDV/3TC/NVP from ≥28 weeks | *CD4≤350/µL:* ZDV/3TC/NVP | *CD4≤350/µL:* ZDV/3TC/NVP through breastfeeding | *CD4≤350/µL*:  ZDV x 7 days (28 days if <4 weeks of antenatal ART) |
|  | *CD4>350/µL:*  None | *CD4>350/µL:*  sdNVP | *CD4>350/µL:*  None | *CD4>350/µL:*  sdNVP |
| **Option A** | *CD4≤350/µL or WHO Stage 3-4:* ZDV/3TC/NVP from >14 weeks | *CD4≤350/µL:* ZDV/3TC/NVP | *CD4≤350/µL:* ZDV/3TC/NVP through breastfeeding | *CD4≤350/µL*:  NVP x 6 weeks |
|  | *CD4>350/µL:*  ZDV from ≥14 weeks | *CD4>350/µL:* sdNVP + ZDV/3TC (if <4 weeks ZDV) | *CD4>350/µL:* ZDV/3TC x 7days | *CD4>350/µL:*  Daily infant NVP until complete cessation of breastfeeding |
| **Option B** | *CD4≤350/µL or WHO Stage 3-4:* ZDV/3TC/NVP from ≥14 weeks | *CD4≤350/µL or WHO Stage 3-4:* ZDV/3TC/NVP | *CD4≤350/µL or WHO Stage 3-4:* ZDV/3TC/NVP lifelong | *CD4≤350/µL or WHO Stage 3-4:* NVP x 6 weeks |
|  | *CD4>350/µL:* ZDV/3TC/NVP from ≥14 weeks | *CD4>350/µL:* ZDV/3TC/NVP | *CD4>350/µL:* ZDV/3TC/NVP through breastfeeding | *CD4>350/µL:* NVP x 6 weeks |

**Supplemental Table 1, continued.**

| **PMTCT**  **Strategy** | **Antenatal**  **(maternal)** | **Intrapartum**  **(maternal)** | **Postpartum**  **(maternal)** | **Neonatal**  **(breastfeeding infant)** |
| --- | --- | --- | --- | --- |
| **Option B+** | *Regardless of CD4 or disease stage:* ZDV/3TC/NVP from ≥14 weeks | *Regardless of CD4 or disease stage:* ZDV/3TC/NVP | *Regardless of CD4 or disease stage:* ZDV/3TC/NVP lifelong | *Regardless of CD4 or disease stage:* NVP x 6 weeks |

**ARVs:** antiretroviral drugs; **sdNVP**: single-dose nevirapine; **NVP**: nevirapine; **ZDV**: zidovudine; **3TC**: lamivudine

**Supplemental Table 2**. Additional input parameters for a model of mother-to-child transmission and postnatal maternal survival in Zimbabwe.

| **Variable** | **Value** |  | | **Data sources** |
| --- | --- | --- | --- | --- |
| **Baseline maternal cohort characteristics** | |  | |  |
| Distribution of initial HIV RNA (% total) | |  | | Cape Town AIDS Cohort |
| >100,000 copies/ml | 43 |  | |  |
| 30,001-100,000 copies/ml | 28 |  | |  |
| 10,001-30,000 copies/ml | 18 |  | |  |
| 3,001-10,000 copies/ml | 8 |  | |  |
| 501-3,000 copies/ml | 2 |  | |  |
| ≤ 500 copies/ml | 1 |  | |  |
| **Natural history of maternal HIV disease** | |  | |  |
| Mean monthly decrease in CD4/µL by HIV RNA | |  | | Multicenter AIDS Cohort Study |
| >30,000 copies/ml | 6.4 |  | |  |
| 10,001-30,000 copies/ml | 5.4 |  | |  |
| 3,001-10,000 copies/ml | 4.6 |  | |  |
| 501-3,000 copies/ml | 3.7 |  | |  |
| 0-500 copies/ml | 3.0 |  | |  |
| Monthly risk of severe opportunistic infections (%, range by CD4 count) | | | Cape Town AIDS Cohort | |
| WHO stage III-IV |  |  | |  |
| Visceral | 0.00-1.52 |  | |  |
| Non-visceral | 0.02-2.26 |  | |  |
| Non-specific | 0.00-0.71 |  | |  |
| Bacterial infection | 0.03-0.71 |  | |  |
| Tuberculosis | 0.16-1.96 |  | |  |
| Other severe infection | 0.14-1.67 |  | |  |

**Supplemental Table 2, continued**

| **Variable** | **Value** | |  | **Data sources** |
| --- | --- | --- | --- | --- |
| **Natural history of maternal HIV disease, continued** | | |  |  |
| Monthly risk of other clinical conditions (%, range by CD4 count) | | |  | Cape Town AIDS Cohort |
| Mild fungal infection | 1.76-3.14 | |
| Other mild infection | 2.33-2.67 | |
| Monthly risk of death from severe opportunistic infections (%) | | | | Cape Town AIDS Cohort |
| WHO stage III-IV |  | |  |
| Visceral | 9.21 | |  |
| Non-visceral | 2.38 | |  |
| Non-specific | 20.00 | |  |
| Bacterial infection | 2.94 | |  |
| Tuberculosis | 1.82 | |  |
| Other severe infection | 6.67 | |  |
| Monthly risk of HIV-related death (%, range by CD4 count) | | |  | Cape Town AIDS Cohort |
| No history of opportunistic infection | | 0.00-4.02 |  |
| With history of opportunistic infection | | 0.00-9.53 |  |
| Monthly risk of death from other clinical conditions (%) | | |  | Cape Town AIDS Cohort |
| Mild fungal infection | 0.54 | |  |
| Other mild infection | 0.39 | |  |
| Relative risk reduction on any ART regimen (%, range by CD4) | | | | Cotrimo-CI, ANRS 1203 |
| HIV-related death | 55-96 | |  |
| Acute opportunistic infections | 0-32 | |  |

**Supplemental Table 2, continued.**

| **Variable** | **Value** | |  | **Data sources** |
| --- | --- | --- | --- | --- |
| **Maternal trimethoprim-sulfamethoxazole effects** | | |  |  |
| % Reduction in probability of infection |  | |  | Cotrimo-CI |
| Mild bacterial diseases | 48.79 | |  |  |
| Invasive bacterial diseases | 49.81 | |  |  |
| WHO stage III-IV visceral diseases and other severe events | 17.88 | |  |  |
| Toxicity risk (%, one-time risk) |  | |  | Cotrimo-CI |
| Minor toxicity | 18.24 | |  |  |
| Major toxicity | 6.72 | |  |  |
| Maternal antiretroviral therapy |  | |  |  |
| **Maternal ART effects** | | |  |  |
| Efficacy (% HIV RNA suppression at 24 weeks) ; gain in CD4/µL at 24 weeks on suppressive ART; yearly risk (%) of virologic failure >24 weeks after initiation) | | | | |
| 1^st^-line NVP/ZDV/3TC initiated in pregnancy | | 90%; 148;17.45% |  |  |
| 1st-line NVP/d4T/3TC post-partum | |  |  |  |
| With sdNVP exposure | | 85%; 148;17.45% |  | OCTANE trial ; |
| Without sdNVP exposure or if sdNVP followed short-course ZDV | | 90%; 148;17.45% |  |  |
| 2nd-line LPV/r/TDF/FTC | | 72%; 148;17.45% |  |  |

WHO: World Health Organization; NVP: nevirapine; d4T: stavudine; 3TC: lamivudine; ZDV: zidovudine; LPV/r: lopinavir/ritonavir; TDF: tenofovir; FTC: emtricibine; scZDV: short-course zidovudine.

**Supplemental Table 3. Internal and external model validationa**

| **I. Infant and childhood mortality** | | | | |
| --- | --- | --- | --- | --- |
|  | **1-year mortality** | | **2-year mortality** | |
|  | **Published risks (%)** | **Model results (%)** | **Published risks (%)** | **Model results (%)** |
| HIV-unexposed | **5.4**  1.9-9.0 | 5.37 | **5.9**  2.9-12.8 | 5.94 |
| HIV-exposed/ uninfected | **7.4**  7.2-7.4 | 7.43 | **9.2**  7.6-16.6 | 9.23 |
| IU/IP-infected, no ART | **51.0**  20.0-58.6 | 50.90 | **65.0**  53.0-67.5 | 64.93 |
| PP-infected, no ART | **24.0**  6.0-26.0 | 23.94 | **38.0**  33.2-52.5 | 37.92 |

**Supplemental Table** 3, continued.

| **II. HIV Transmission Risks** | | | | | | |
| --- | --- | --- | --- | --- | --- | --- |
| **PMTCT regimen and maternal CD4 stratum** | **HIV infection risk at**  **4-6 weeks of age (%)** | | **Cumulative HIV infection risk at**  **18 months of age (%)** | | **HIV infection risk at 18 months of age among infants uninfected at 4-6 wks (%)** | |
|  | **Published risks** | **Model results** | **Published risks** | **Model results** | **Published risks** | **Model results** |
| **No antenatal ARVs**  <350/µL | **27.3**  <500: 32.0 | 26.1 | <200: 24m 33.3  <350: 52.1  <500: 38.0-41.3 | 38.8 | **20.0** | 18.2 |
| ≥350/µL | **17.5**  >500: 19.3 | 16.7 | 350-500: 27.7  >500: 21.2  24m 14.1-22.0 | 21.8 | **6.8** | 6.4 |
| Combined CD4 strata^a^ | 15.3-24.8 | 20.1 | 200-500 18m: 27.9  15-24m: 22.2-36.7 | 27.9 | **6.9 (EBF), 11.2 (MBF)**  6.3-21.0 | 10.7 |

**Supplemental Table** 3, continued.

| **II. HIV Transmission Risks, continued** | | | | | | |
| --- | --- | --- | --- | --- | --- | --- |
| **PMTCT regimen and maternal CD4 stratum** | **HIV infection risk at**  **4-6 weeks of age (%)** | | **Cumulative HIV infection risk at**  **18 months of age (%)** | | **HIV infection risk at 18 months of age among infants uninfected at 4-6 wks (%)** | |
|  | **Published risks** | **Model results** | **Published risks** | **Model results** | **Published risks** | **Model results** |
| **sdNVP**  <350/µL | **17.6**  <200: 10.7-23.1  200-350: 5.6-14.9 | 16.9 | <200: 23.4  200-350: 19.5 | 31.2 ^b^ | <200: 14.2  200-350: 14.7  To 4m: 15.8  **20.0 (no antenatal** **ARVs)** | 18.2 |
| ≥350/µL | **7.3**  >350: 3.3 , >500: 8.4 , 350-500: 5.8 | 7.0 | 8.9 | 12.7 ^b^ | 5.8  To 4m: 4.3  **6.8 (no antenatal ARVs)** | 6.4 |
| Combined CD4 strata | 11.2-21.8 | 10.5 | 15.7-25.8 | 19.4 ^b^ | 4.4-10.6 | 10.7 |

**Supplemental Table** 3, continued.

| **II. HIV Transmission Risks, continued** | | | | | | |
| --- | --- | --- | --- | --- | --- | --- |
| **PMTCT regimen and maternal CD4 stratum** | **HIV infection risk at**  **4-6 weeks of age (%)** | | **Cumulative HIV infection risk at**  **18 months of age (%)** | | **HIV infection risk at 18 months of age among infants uninfected at 4-6 wks (%)** | |
|  | **Published risks** | **Model results** | **Published risks** | **Model results** | **Published risks** | **Model results** |
| **Option A**  <350/µL | **13.6** | n/a | (14 wk NVP): 15.4-15.5 | n/a | (14wk NVP): 12.8-13.4 | n/a |
| ≥350/µL | **3.6**  >200 RF: 8.3 | 3.5 | (14 wk NVP): 6.2  >250 (at 7m): 6.0 | 6.8 | (14wk NVP): 5.1  >250 (at 7m): 1.7  Median CD4 370-430 (at 6m):  0.6, **1.1,** 1.2-4.5 | 3.7 |
| Combined CD4 strata | 4.6-14.7  200-500: 4.8 | n/a | n/a | n/a | n/a | n/a |

**Supplemental Table** 3, continued.

| **II. HIV Transmission Risks, continued** | | | | | | |
| --- | --- | --- | --- | --- | --- | --- |
| **PMTCT regimen and maternal CD4 stratum** | **HIV infection risk at**  **4-6 weeks of age (%)** | | **Cumulative HIV infection risk at**  **18 months of age (%)** | | **HIV infection risk at 18 months of age among infants uninfected at 4-6 wks (%)** | |
|  | **Published risks** | **Model results** | **Published risks** | **Model results** | **Published risks** | **Model results** |
| **Option B/B+**  <350/µL | **3.3**  <200: 0.6-3.7 | 3.3 | <200: 6m 5.6, 12m 7.5, 18m 7.5  200-350: 6m 5.5, 12m 6.1 | 8.4 | <200: 6m 0-2.0 ,  18m 0-3.9 | 5.5 |
| ≥350/µL | **1.0**  >200: 0.4-4.1  >250: 3.8 | 1.0 | 350-500: 6m 4.1, 12m 4.9  >200: 12m 6.2  >250 7m: 8.2 | 4.0 | >200: 6m 0-0.7%  >250: 6m **1.1 -** 1.3 , 7m 2.9  >250: 12m 0.5-1.8 | 3.1 |
| Combined CD4 strata | 1.2-3.3 | 1.8 | 200-500 6m 4.9, 18m 5.5 | 5.6 | 200-500: 6m **1.7** , (base case input for <350) | 4.0 |

a. Internal validation compares the degree to which model output mirrors the data used as model inputs (highlighted in boldface type and further detailed in Manuscript Table 1). External validation compares model outputs to other published cohorts. To validate against published data from clinical trials, these analyses include infants of women HIV-infected at first ANC visit who are identified as HIV-infected and receive only the specified PMTCT regimen. Model results are projected at 4-6 weeks of age or 18 months of age, for 1) women with CD4 ≤350/µL, 2) women with CD4 >350/µL, and 3) a cohort of women similar to the ZVITAMBO cohort in Zimbabwe, of whom 36% have CD4 ≤350/µL. Where published reports were not available to match these time points and CD4-stratified cohorts exactly, the closest available data are presented for comparison.

b. HIV infection risks at 18 months of age following receipt of sdNVP reflect modeled risk of breastfeeding transmission equal to that of the No ARV strategy after 6 weeks of age.

EBF: exclusive breastfeeding; MBF: mixed breastfeeding; RF: replacement feeding; m: months of age; sdNVP: single-dose nevirapine; ZDV: zidovudine; n/a: not applicable or not available.

**Supplemental Table 4. Intermediate (CEPAC model) results^a^: Maternal outcomes after delivery, stratified by HIV disease severity and access to postnatal HIV care**

| **Maternal HIV status and access to care at 6 weeks postpartum** | **Life expectancy (years, from delivery)** | **2-year**  **survival (%)** |
| --- | --- | --- |
| **ART eligible ^b^** |  |  |
| On ART in pregnancy, continue lifelong | 14.6 | 96.1 |
| In postnatal HIV care, no sdNVP exposure | 14.6 | 96.0 |
| In postnatal HIV care, after sdNVP exposure | 14.4 | 95.9 |
| Not in postnatal HIV care,^c^ no sdNVP exposure | 8.5 | 81.8 |
| Not in postnatal HIV care,^c^ after sdNVP exposure | 8.4 | 81.8 |
| **Not ART eligible ^b^** |  |  |
| On ART in pregnancy, continue lifelong | 17.7 | 97.8 |
| Initiate ART at 6-week PP visit, after sdNVP exposure, continue lifelong | 17.5 | 97.8 |
| On ART in pregnancy, interrupt at 18 months (weaning) | 16.3 | 97.1 |
| Initiate ART at 6-week PP visit, after sdNVP exposure, interrupt at 18 months (weaning) | 16.0 | 97.1 |
| In postnatal HIV care, no sdNVP exposure | 16.6 | 94.2 |
| In postnatal HIV care, after sdNVP exposure | 16.4 | 94.2 |
| Not in postnatal HIV care,^c^ no sdNVP exposure | 11.8 | 94.1 |
| Not in postnatal HIV care,^c^ after sdNVP exposure | 11.7 | 94.1 |

**Supplemental Table** 4, continued.

| **Maternal HIV status and access to care at 6 weeks postpartum** | **Life expectancy (years, from delivery)** | **2-year**  **survival** |
| --- | --- | --- |
| **Incident infection during pregnancy** | | |
| Initiate ART at 6-week PP visit, no sdNVP exposure,  continue lifelong | 18.8 | 98.0 |
| Initiate ART at 6-week PP visit, after sdNVP exposure, continue lifelong | 18.6 | 97.9 |
| Initiate ART at 6-week PP visit, no sdNVP exposure,  interrupt at 18 months (weaning) | 17.2 | 97.3 |
| Initiate ART at 6-week PP visit, after sdNVP exposure, interrupt at 18 months (weaning) | 16.9 | 97.3 |
| In postnatal HIV care, no sdNVP exposure | 17.4 | 95.5 |
| In postnatal HIV care, after sdNVP exposure | 17.2 | 95.5 |
| Not in postnatal HIV care,^c^ no sdNVP exposure | 13.1 | 95.5 |
| Not in postnatal HIV care,^c^ after sdNVP exposure | 13.0 | 95.5 |
| **HIV-negative through pregnancy and breastfeeding** | | |
|  | 49.9 | 99.5 |

a. CEPAC model outputs were intermediate results, used as inputs to the MTCT model.

b. ART eligibility was defined as CD4 ≤350/µL, or WHO stage 3-4 disease ,

c. Women who do not link to postnatal HIV care (defined as linkage by 6 weeks postpartum) are assumed to present to HIV care upon development of a severe OI.

Supplemental Table 5. Model-based outcomes of strategies to prevent mother-to-child HIV transmission: General Zimbabwean population

|  | **Model results ^a^** | | | |
| --- | --- | --- | --- | --- |
| **Pediatric outcomes** |  |  |  | |
|  | **Risk of HIV infection**  **at birth (%; number of infected infants)** | **Risk of HIV infection**  **at 18 months (%; number of infected infants)** | | **2-year**  **survival (%)** |
| No **antenatal** ARVs | 3.4%; 13,530 | 4.5%; 17,530 | | 89.0 |
| Single-dose NVP | 1.9%; 7,650 | 3.1%; 12,070 | | 89.5 |
| Option A | 1.4%; 5,370 | 2.4%; 9,280 | | 89.8 |
| Option B | 1.1%; 4,230 | 2.1%; 8,080 | | 90.0 |
| Option B+ | 1.1%: 4,230 | 2.1%; 8,080 | | 90.0 |
| **Maternal outcomes after delivery** | | | | |
|  | **2-year**  **survival (%)** | **5-year**  **survival (%)** | | **Life expectancy (years)** |
| No **antenatal** ARVs | 97.7 | 94.9 | | 43.1 |
| Single-dose NVP | 97.7 | 94.8 | | 43.1 |
| Option A | 97.7 | 94.9 | | 43.1 |
| Option B | 97.9 | 94.9 | | 43.1 |
| Option B+ | 98.0 | 95.2 | | 43.2 |

a. Results are shown for all pregnant women in Zimbabwe (392,460 women/year), with HIV prevalence of 16% and HIV incidence of 0.96%/year.

***Supplemental Table 6. Sensitivity analyses: impact of key model parameters on outcomes of PMTCT strategies in Zimbabwe***

|  | **Risk of pediatric HIV infection at birth (%)** | | **Risk of pediatric HIV infection at 18m (%)** | | | **2-year pediatric**  **survival (%)** | | | | **Maternal LE (years, undiscounted)** | |
| --- | --- | --- | --- | --- | --- | --- | --- | --- | --- | --- | --- |
| **A. Base case results (also shown in Manuscript Table 3) ^a^** | | | | | | | | | | | |
| No **antenatal** ARVs | 20.1 | | 25.8 | | | | 78.4 | | | 14.0 | |
| Single-dose NVP | 10.8 | | 17.2 | | | | 81.9 | | | 13.8 | |
| Option A | 7.2 | | 12.8 | | | | 83.5 | | | 14.0 | |
| Option B | 5.4 | | 10.9 | | | | 84.9 | | | 13.9 | |
| Option B+ | 5.4 | | 10.9 | | | | 84.9 | | | 14.4 | |
| **B. Clinical parameters impacting model results ^b^** | | | | | | | | | | | |
| **Prevalence of maternal ART eligibility (base case=36%)** | | | | | | | | | | | |
| **Proportion ART-eligible** | **0%** | **100%** | | **0%** | **100%** | | **0%** | **100%** | **0%** | | **100%** |
| No **antenatal** ARVs | 16.7 | 26.1 | | 21.8 | 32.9 | | 79.8 | 75.9 | 14.8 | | 12.4 |
| Single-dose NVP | 8.7 | 14.6 | | 14.0 | 22.4 | | 82.7 | 80.4 | 14.7 | | 12.3 |
| Option A | 5.9 | 9.4 | | 10.0 | 17.7 | | 84.0 | 82.6 | 14.8 | | 12.4 |
| Option B | 4.0 | 7.8 | | 7.9 | 16.2 | | 85.7 | 83.4 | 14.7 | | 12.4 |
| Option B+ | 4.0 | 7.8 | | 7.9 | 16.2 | | 85.7 | 83.4 | 15.6 | | 12.4 |

***Supplemental Table 6, continued***

|  | **Risk of pediatric HIV infection at birth (%)** | | **Risk of pediatric HIV infection at 18m (%)** | | | **2-year pediatric**  **survival (%)** | | | | **Maternal LE (years, undiscounted)** | |
| --- | --- | --- | --- | --- | --- | --- | --- | --- | --- | --- | --- |
| **B. Clinical parameters impacting model results, continued ^b^** | | | | | | | | | | | |
| **Difference in 1^st^-line maternal ART efficacy following sdNVP exposure (base case: no sdNVP=90%, following sdNVP=85%, ∆=5%)** | | | | | | | | | | | |
| **∆ RNA<400 copies/ml, vs. no NVP exposure** | **n/a** | **n/a** | | **n/a** | **n/a** | | **n/a** | **n/a** | **0%** | | **16%** |
| No **antenatal** ARVs |  |  | |  |  | |  |  | 14.0 | | 14.0 |
| Single-dose NVP |  |  | |  |  | |  |  | 14.0 | | 13.6 |
| Option A |  |  | |  |  | |  |  | 14.0 | | 14.0 |
| Option B |  |  | |  |  | |  |  | 14.0 | | 13.9 |
| Option B+ |  |  | |  |  | |  |  | 14.4 | | 14.4 |
| **Best-case and worst-case MTCT risks (base case: see Manuscript Table 1)** | | | | | | | | | | | |
|  | **Best-case** | **Worst-case** | | **Best-case** | **Worst-case** | | **Best-case** | **Worst-case** | **n/a** | | **n/a** |
| No **antenatal** ARVs | 14.6 | 23.7 | | 16.6 | 33.0 | | 81.0 | 76.6 |  | |  |
| Single-dose NVP | 5.9 | 15.0 | | 8.1 | 25.4 | | 84.3 | 79.8 |  | |  |
| Option A | 4.7 | 8.6 | | 6.7 | 17.4 | | 84.9 | 82.5 |  | |  |
| Option B | 3.3 | 7.3 | | 4.5 | 17.7 | | 86.3 | 83.5 |  | |  |
| Option B+ | 3.3 | 7.3 | | 4.5 | 17.7 | | 86.3 | 83.5 |  | |  |

|  | **Risk of pediatric HIV infection at birth (%)** | | | **Risk of pediatric HIV infection at 18m (%)** | | | **2-year pediatric**  **survival (%)** | | | | **Maternal LE (years, undiscounted)** | |
| --- | --- | --- | --- | --- | --- | --- | --- | --- | --- | --- | --- | --- |
| **B. Clinical parameters impacting model results, continued ^b^** | | | | | | | | | | | | |
| **Non-HIV-deleted mortality rates (base case: UNAIDS HIV-deleted rates for Zimbabwe)** | | | | | | | | | | | | |
| **Data source** | **n/a** | | **n/a** | | **n/a** | **n/a** | | **n/a** | **n/a** | | **South Africa, HIV-deleted** | **Zimbabwe, non-HIV-deleted** |
| No **antenatal** ARVs |  | |  | |  |  | |  |  | | 14.0 | 11.1 |
| Single-dose NVP |  | |  | |  |  | |  |  | | 13.9 | 11.0 |
| Option A |  | |  | |  |  | |  |  | | 14.0 | 11.1 |
| Option B |  | |  | |  |  | |  |  | | 13.9 | 11.0 |
| Option B+ |  | |  | |  |  | |  |  | | 14.5 | 11.5 |
| **C. Access to care parameters impacting model results ^b^** | | | | | | | | | | | | |
| **Access to antenatal care (ANC, base case=91%)** | | | | | | | | | | | | |
| **Proportion accessing ANC** | | **80%** | **100%** | | **80%** | **100%** | | **80%** | **100%** | **80%** | | **100%** |
| No **antenatal** ARVs | 20.1 | | 20.1 | | 26.0 | 25.7 | | 78.4 | 78.4 | 13.7 | | 14.2 |
| Single-dose NVP | 11.7 | | 10.1 | | 18.2 | 16.4 | | 81.5 | 82.2 | 13.6 | | 14.1 |
| Option A | 8.5 | | 6.1 | | 14.3 | 11.6 | | 82.9 | 83.9 | 13.7 | | 14.2 |
| Option B | 6.9 | | 4.1 | | 12.6 | 9.5 | | 84.2 | 85.5 | 13.6 | | 14.1 |
| Option B+ | 6.9 | | 4.1 | | 12.6 | 9.5 | | 84.2 | 85.5 | 14.1 | | 14.7 |

***Supplemental Table 6, continued***

|  | **Risk of pediatric HIV infection at birth (%)** | | | **Risk of pediatric HIV infection at 18m (%)** | | | | **2-year pediatric**  **survival (%)** | | | **Maternal LE (years, undiscounted)** | | |
| --- | --- | --- | --- | --- | --- | --- | --- | --- | --- | --- | --- | --- | --- |
| **C. Access to care parameters impacting model results, continued ^b^** | | | | | | | | | | | | | |
| **Rate of HIV testing in ANC (base case=87%)** | | | | | | | | | | | | | |
| **Proportion undergoing HIV test in ANC** | | **58%** | **100%** | | **58%** | | **100%** | | **58%** | **100%** | | **58%** | **100%** |
| No **antenatal** ARVs | | 20.1 | 20.1 | | 26.3 | | 25.6 | | 78.3 | 78.5 | | 13.2 | 14.3 |
| Single-dose NVP | | 13.3 | 9.7 | | 20.0 | | 16.0 | | 80.9 | 82.3 | | 13.1 | 14.2 |
| Option A | | 10.9 | 5.6 | | 16.9 | | 10.9 | | 81.9 | 84.2 | | 13.2 | 14.3 |
| Option B | | 9.7 | 3.5 | | 15.6 | | 8.7 | | 82.9 | 85.8 | | 13.1 | 14.2 |
| Option B+ | | 9.7 | 3.5 | | 15.6 | | 8.7 | | 82.9 | 85.8 | | 13.5 | 14.9 |
| **Rate of HIV test result return = 71% (base case=99%)** | | | | | | | | | | | | | |
| No **antenatal** ARVs | | 20.1 | | | | 26.2 | | | 78.3 | | 13.3 | | |
| Single-dose NVP | | 12.9 | | | | 19.6 | | | 81.0 | | 13.2 | | |
| Option A | | 10.3 | | | | 16.3 | | | 82.2 | | 13.3 | | |
| Option B | | 9.0 | | | | 14.9 | | | 83.2 | | 13.2 | | |
| Option B+ | | 9.0 | | | | 14.9 | | | 83.2 | | 13.7 | | |

**Supplemental Table 6, continued**

|  | **Risk of pediatric HIV infection at birth (%)** | | **Risk of pediatric HIV infection at 18m (%)** | | | | **2-year pediatric**  **survival (%)** | | **Maternal LE (years, undiscounted)** | | |
| --- | --- | --- | --- | --- | --- | --- | --- | --- | --- | --- | --- |
| **C. Access to care parameters impacting model results, continued ^b^** | | | | | | | | | | | |
| **Rate of HIV testing and result receipt = 100% (base case=87% test, 99% result receipt)** | | | | | | | | | | | |
| No **antenatal** ARVs | | 20.1 | | | 25.6 | | 78.5 | | 14.4 | | |
| Single-dose NVP | | 9.6 | | | 15.9 | | 82.4 | | 14.2 | | |
| Option A | | 5.4 | | | 10.8 | | 84.2 | | 14.4 | | |
| Option B | | 3.3 | | | 8.6 | | 85.9 | | 14.3 | | |
| Option B+ | | 3.3 | | | 8.6 | | 85.9 | | 14.9 | | |
| **Linkage to postnatal care (base case=79%)** | | | | | | | | | | | |
| **Proportion of mothers linking to postnatal care** | | **n/a** | | **51%** | | **100%** | **51%** | **100%** | | **51%** | **100%** |
| No **antenatal** ARVs | |  | | 26.6 | | 25.3 | 78.3 | 78.5 | | 12.8 | 14.9 |
| Single-dose NVP | |  | | 18.1 | | 16.5 | 81.7 | 82.0 | | 12.7 | 14.7 |
| Option A | |  | | 14.1 | | 11.8 | 83.3 | 83.7 | | 12.8 | 14.9 |
| Option B | |  | | 12.3 | | 9.8 | 84.6 | 85.1 | | 12.7 | 14.8 |
| Option B+ | |  | | 12.3 | | 9.8 | 84.6 | 85.1 | | 13.1 | 15.5 |

**Supplemental Table 6, continued**

|  | **Risk of pediatric HIV infection at birth (%)** | | | **Risk of pediatric HIV infection at 18m (%)** | | **2-year pediatric**  **survival (%)** | | **Maternal LE (years, undiscounted)** | | |
| --- | --- | --- | --- | --- | --- | --- | --- | --- | --- | --- |
| **C. Access to care parameters impacting model results, continued ^b^** | | | | | | | | | | |
| **Full (100%) uptake at each step of the cascade through linkage to maternal postnatal care (base case: see Manuscript Table 1)** | | | | | | | | | | |
| No **antenatal** ARVs | 20.1 | | | 24.7 | | 78.6 | | 15.7 | | |
| Single-dose NVP | 8.8 | | | 14.1 | | 82.9 | | 15.6 | | |
| Option A | 3.4 | | | 7.4 | | 85.3 | | 15.7 | | |
| Option B | 1.8 | | | 5.6 | | 86.8 | | 15.6 | | |
| Option B+ | 1.8 | | | 5.6 | | 86.8 | | 16.5 | | |
| **Access to pediatric HIV care and ART (base case=36%)** | | | | | | | | | | |
| **Proportion initiating ART** | **n/a** | **n/a** | **n/a** | | **n/a** | **0%** | **100%** | | **n/a** | **n/a** |
| No **antenatal** ARVs |  |  |  | |  | 74.2 | 86.0 | |  |  |
| Single-dose NVP |  |  |  | |  | 79.4 | 86.4 | |  |  |
| Option A |  |  |  | |  | 81.7 | 86.7 | |  |  |
| Option B |  |  |  | |  | 83.5 | 87.4 | |  |  |
| Option B+ |  |  |  | |  | 83.5 | 87.4 | |  |  |

**Supplemental Table 6, continued**

| **D. Model input parameters not substantially changing model results (range examined) ^b^** |
| --- |
| Maternal age at first ANC visit (20-30 years) |
| Probability of delivery at home (0 - 100%) |
| Maternal mortality during pregnancy (0-2%) |
| Probability of live birth (1-5%) |
| Difference in survival for HIV-infected infants exposed and not exposed to sdNVP (0-10%) |
| Non-HIV-related infant mortality rates |
| Impact of maternal mortality on infant survival (relative risk, 1-6) |
| Rate of cell CD4 decline after ART interruption (75-139 cells over 6 months post-interruption) |
| Sensitivity of clinical assessment for ART eligibility (20%– 50%). Base case value of 36% is reported sensitivity for CD4 <200/ µL; reported value for CD4 <350/µL is 20%. |

ANC: antenatal care, NVP: nevirapine, sdNVP: single-dose nevirapine; MTCT: mother-to-child transmission; ART: antiretroviral therapy

a. Base-case results and all sensitivity analyses are shown for a cohort of chronically HIV-infected, pregnant women in Zimbabwe, as in Manuscript Table 3 (mean age 24.0 years, mean CD4 451 cells/µL).

b. Substantial change in model results was defined as: 1) a change in the order of the results for each strategy, or 2) a >10% relative change in the difference in outcomes for each strategy.

**Supplemental Table 7. Determination of "substantial" change in model results after variation of key model parameters in sensitivity analyses.**

| **Base case results (64% of women with CD4 >350/µL)** | | | **Sensitivity analysis: all women with CD4 >350/µL** | | | **Proportional change in delta between regimen and next-best regimen (before rounding)** |
| --- | --- | --- | --- | --- | --- | --- |
| **Regimen** | **Maternal LE (years)** | **Delta (vs. next best regimen)** | **Regimen** | **Maternal LE (years)** | **Delta (vs. next best regimen)** |
| sdNVP | 13.8 | -- | sdNVP | 14.7 | -- | -- |
| Option B | 13.9 | 0.1 | Option B | 14.7 | 0.0 | -0.06 |
| Option A | 14.0 | 0.1 | Option A | 14.8 | 0.1 | 0.57 |
| No antenatal ARVs | 14.0 | 0.0 | No antenatal ARVs | 14.8 | 0.0 | 0.37 |
| Option B+ | 14.5 | 0.5 | Option B+ | 15.6 | 0.8 | 0.56 |

Example of identification of a "substantial" change in results after variation in a model input parameter. In the base case analysis, projected maternal life expectancies for each regimen are as shown in the left section, second column. The differences in LE between each regimen and the next best one are shown in the left section, third column. In a sensitivity analysis in which all women had CD4 cell counts ≥350/µL (as reported above in Supplemental Table 6), the projected life expectancies were higher for all regimens. Because the order of the outcomes for the five regimens did not change, the first definition of a "substantial" change in results was not met. However, the differences between life expectancy for each regimen compared to the next best regimen changed by >10% of the base-case delta for most pairs of regimens compared. Although there was a relative decrease of only 6% for Option B vs. sdNVP, there were relative increases of 57% for Option A vs. Option B, 37% for no antenatal ARVs vs Option A, and 56% for Option B+ vs. Option B. Maternal CD4 cell count therefore met the definition of a parameter leading to a substantial change in model results, due to a >10% relative change in the difference between projected outcomes for each strategy. **LE**: life expectancy.

**REFERENCES:**

1. Ministry of Health and Child Welfare Zimbabwe. Maternal and Perinatal Mortality Study, 2007.

2. Ministry of Health Zimbabwe. National HIV Estimates, 2009.

3. Barker PM, Mphatswe W, Rollins N. Antiretroviral drugs in the cupboard are not enough: The impact of health systems' performance on mother-to-child transmission of HIV. *J Acquir Immune Defic Syndr* 2010,56:e45-48.

4. Walensky RP, Paltiel AD, Losina E*, et al.* The survival benefits of AIDS treatment in the United States. *J Infect Dis* 2006,194:11-19.

5. Freedberg KA, Losina E, Weinstein MC*, et al.* The cost effectiveness of combination antiretroviral therapy for HIV disease. *N Engl J Med* 2001,344:824-831.

6. Paltiel AD, Weinstein MC, Kimmel AD*, et al.* Expanded screening for HIV in the United States--an analysis of cost-effectiveness. *N Engl J Med* 2005,352:586-595.

7. Goldie SJ, Yazdanpanah Y, Losina E*, et al.* Cost-effectiveness of HIV treatment in resource-poor settings--the case of Côte d'Ivoire. *N Engl J Med* 2006,355:1141-1153.

8. Walensky RP, Wood R, Weinstein MC*, et al.* Scaling up antiretroviral therapy in South Africa: the impact of speed on survival. *J Infect Dis* 2008,197:1324-1332.

9. Walensky RP, Wolf LL, Wood R*, et al.* When to start antiretroviral therapy in resource-limited settings. *Ann Intern Med* 2009,151:157-166.

10. El-Sadr WM, Lundgren JD, Neaton JD*, et al.* CD4+ count-guided interruption of antiretroviral treatment. *N Engl J Med* 2006,355:2283-2296.

11. Danel C, Moh R, Minga A*, et al.* CD4-guided structured antiretroviral treatment interruption strategy in HIV-infected adults in west Africa (Trivacan ANRS 1269 trial): a randomised trial. *Lancet* 2006,367:1981-1989.

12. Mellors JW, Munoz A, Giorgi JV*, et al.* Plasma viral load and CD4+ lymphocytes as prognostic markers of HIV-1 infection. *Ann Intern Med* 1997,126:946-954.

13. Ministry of Health and Child Welfare Zimbabwe: National Drug and Therapeutics Policy Advisory Committee (NDTPAC) & AIDS and TB Unit. Guidelines for Antiretroviral Therapy in Zimbabwe, 2009.

14. World Health Organization. Antiretroviral therapy for HIV infection in adults and adolescents - Recommendations for a public health approach (2010 version). Accessed on August 24, 2010 at http://www.who.int/hiv/pub/arv/adult2010/en/index.html.

15. Losina E, Yazdanpanah Y, Deuffic-Burban S*, et al.* The independent effect of highly active antiretroviral therapy on severe opportunistic disease incidence and mortality in HIV-infected adults in Côte d'Ivoire. *Antivir Ther* 2007,12:543-551.

16. Chigwedere P, Seage GR, Lee TH, Essex M. Efficacy of antiretroviral drugs in reducing mother-to-child transmission of HIV in Africa: a meta-analysis of published clinical trials. *AIDS Res Hum Retroviruses* 2008,24:827-837.

17. Dabis F, Bequet L, Ekouevi DK*, et al.* Field efficacy of zidovudine, lamivudine and single-dose nevirapine to prevent peripartum HIV transmission. *AIDS* 2005,19:309-318.

18. Kuhn L, Aldrovandi GM, Sinkala M*, et al.* Potential impact of new WHO criteria for antiretroviral treatment for prevention of mother-to- child HIV transmission. *AIDS* 2010,24:1374-1377; additional data at http://www.hivpresentation.com/index.cfm?vId=1375BB1328A1344-1423A-F1376F1377-C1370DC1376EC1268CFF1357&cID=1375C1373B1824B-1423A-F1376F1377-C1372E1378CE1379BBF1379B1372D1392&show=slide.

19. World Bank. Population data, 2009. Accessed on February 22, 2011 at http://data.worldbank.org/indicator/SP.POP.TOTL.

20. Index Mundi. Zimbabwe birth rate, 2009. Accessed on February 22, 2011 at http://www.indexmundi.com/zimbabwe/birth_rate.html.

21. Fawzi W, Msamanga G, Spiegelman D*, et al.* Transmission of HIV-1 through breastfeeding among women in Dar es Salaam, Tanzania. *J Acquir Immune Defic Syndr* 2002,31:331-338.

22. Coutsoudis A, Pillay K, Kuhn L*, et al.* Method of feeding and transmission of HIV-1 from mothers to children by 15 months of age: prospective cohort study from Durban, South Africa. *AIDS* 2001,15:379-387.

23. Nduati R, John G, Mbori-Ngacha D*, et al.* Effect of breastfeeding and formula feeding on transmission of HIV-1: a randomized clinical trial. *JAMA* 2000,283:1167-1174.

24. Petra Study Team. Efficacy of three short-course regimens of zidovudine and lamivudine in preventing early and late transmission of HIV-1 from mother to child in Tanzania, South Africa, and Uganda (Petra study): a randomised, double-blind, placebo-controlled trial. *Lancet* 2002,359:1178-1186.

25. Guay LA, Musoke P, Fleming T*, et al.* Intrapartum and neonatal single-dose nevirapine compared with zidovudine for prevention of mother-to-child transmission of HIV-1 in Kampala, Uganda: HIVNET 012 randomised trial. *Lancet* 1999,354:795-802.

26. Thistle P, Spitzer RF, Glazier RH*, et al.* A randomized, double-blind, placebo-controlled trial of combined nevirapine and zidovudine compared with nevirapine alone in the prevention of perinatal transmission of HIV in Zimbabwe. *Clin Infect Dis* 2007,44:111-119.

27. Thior I, Lockman S, Smeaton LM*, et al.* Breastfeeding plus infant zidovudine prophylaxis for 6 months vs formula feeding plus infant zidovudine for 1 month to reduce mother-to-child HIV transmission in Botswana: a randomized trial: the Mashi Study. *JAMA* 2006,296:794-805.

28. Palombi L, Marazzi MC, Voetberg A, Magid NA. Treatment acceleration program and the experience of the DREAM program in prevention of mother-to-child transmission of HIV. *AIDS* 2007,21 Suppl 4:S65-71.

29. Thomas T, Masamba R, Ndivo R , Zeh C, Borkowf C, Thigpen M, De Cock K, Amornkul P, Greenberg A , Fowler M, and Kisumu Breastfeeding Study Team. 45aLB: Prevention of mother-to-child transmission of HIV-1 among breastfeeding mothers using HAART: The Kisumu Breastfeeding Study, Kisumu, Kenya, 2003–2007. Conference on Retroviruses and Opportunistic Infections, Boston, 2008, http://www.retroconference.org/2008/Abstracts/33397.htm.

30. Kilewo C, Karlsson K, Ngarina M*, et al.* Prevention of mother-to-child transmission of HIV-1 through breastfeeding by treating mothers with triple antiretroviral therapy in Dar es Salaam, Tanzania: the Mitra Plus study. *J Acquir Immune Defic Syndr* 2009,52:406-416.

31. Iliff PJ, Piwoz EG, Tavengwa NV*, et al.* Early exclusive breastfeeding reduces the risk of postnatal HIV-1 transmission and increases HIV-free survival. *AIDS* 2005,19:699-708.

32. Chasela CS, Hudgens MG, Jamieson DJ*, et al.* Maternal or infant antiretroviral drugs to reduce HIV-1 transmission. *N Engl J Med* 2010,362:2271-2281.

33. Shapiro RL, Hughes MD, Ogwu A*, et al.* Antiretroviral regimens in pregnancy and breast-feeding in Botswana. *N Engl J Med* 2010,362:2282-2294.

34. Mofenson L, Taha T, Li Q, Kumwenda J, Kafulafula G, Fowler MG, Hoover DR, Thigpen M, Kumwenda N, and the PEPI Malawi Study Group. TUPEC053: Infant extended antiretroviral (ARV) prophylaxis is effective in preventing postnatal mother-to-child HIV transmission (MTCT) at all maternal CD4 counts International AIDS Society, Cape Town, South Africa, 2009, http://www.ias2009.org/pag/Abstracts.aspx?AID=1251.

35. Kesho Bora Study Group. Triple antiretroviral compared with zidovudine and single-dose nevirapine prophylaxis during pregnancy and breastfeeding for prevention of mother-to-child transmission of HIV-1 (Kesho Bora study): a randomised controlled trial. *Lancet Infect Dis* 2011.

36. Kuhn L, Sinkala M, Kankasa C*, et al.* High uptake of exclusive breastfeeding and reduced early post-natal HIV transmission. *PLoS One* 2007,2:e1363.

37. Leroy V, Newell ML, Dabis F*, et al.* International multicentre pooled analysis of late postnatal mother-to-child transmission of HIV-1 infection. Ghent International Working Group on Mother-to-Child Transmission of HIV. *Lancet* 1998,352:597-600.

38. Vyankandondera J, Luchters S, Hassink E. N°LB7: Reducing risk of HIV-1 transmission from mother to infant through breastfeeding using antiretroviral prophylaxis in infants (SIMBA-study). International AIDS Society, Paris, France, 2003,

39. Marinda E, Humphrey JH, Iliff PJ*, et al.* Child Mortality According to Maternal and Infant HIV Status in Zimbabwe. *Pediatr Infect Dis J* 2007,26:519-526.

40. Newell ML, Coovadia H, Cortina-Borja M*, et al.* Mortality of infected and uninfected infants born to HIV-infected mothers in Africa: a pooled analysis. *Lancet* 2004,364:1236-1243.

41. Marston M, Becquet R, Zaba B*, et al.* Net survival of perinatally and postnatally HIV-infected children: a pooled analysis of individual data from sub-Saharan Africa. *Int J Epidemiol* 2011.

42. Brahmbhatt H, Kigozi G, Wabwire-Mangen F*, et al.* Mortality in HIV-infected and uninfected children of HIV-infected and uninfected mothers in rural Uganda. *J Acquir Immune Defic Syndr* 2006,41:504-508.

43. Ciaranello A, Lockman S, Freedberg KA, Hughes M, Chu J, Currier J, Wood R, Holmes CB, Pillay S, Conradie F, McIntyre J, Losina E, Walensky RP. First-line antiretroviral therapy after single-dose nevirapine exposure in South Africa: A cost-effectiveness analysis of the OCTANE trial. *AIDS* 2011,25:479-492.

44. UNAIDS/WHO Working Group on Global HIV/AIDS and STI Surveillance. Epidemiological Fact Sheet on HIV and AIDS Zimbabwe 2008 Update. 2009.Accessed on March 24, 2010 at http://apps.who.int/globalatlas/predefinedReports/EFS2008/full/EFS2008_ZW.pdf.

45. Crampin AC, Floyd S, Glynn JR*, et al.* The long-term impact of HIV and orphanhood on the mortality and physical well-being of children in rural Malawi. *AIDS* 2003,17:389-397.

46. Nakiyingi JS, Bracher M, Whitworth JA*, et al.* Child survival in relation to mother's HIV infection and survival: evidence from a Ugandan cohort study. *AIDS* 2003,17:1827-1834.

47. Zaba B, Whitworth J, Marston M*, et al.* HIV and mortality of mothers and children: evidence from cohort studies in Uganda, Tanzania, and Malawi. *Epidemiology* 2005,16:275-280.

48. Lockman S, Shapiro RL, Smeaton LM*, et al.* Response to antiretroviral therapy after a single, peripartum dose of nevirapine. *N Engl J Med* 2007,356:135-147.

49. Jourdain G, Ngo-Giang-Huong N, Le Coeur S*, et al.* Intrapartum exposure to nevirapine and subsequent maternal responses to nevirapine-based antiretroviral therapy. *N Engl J Med* 2004,351:229-240.

50. Palumbo P, Violara A, Lindsey J, Hughes M, Jean-Philippe P, Mofenson L, Purdue L, Eshleman S, and the IMPAACT P1060 Study Team,. LBPEB12: Nevirapine (NVP) vs lopinavir-ritonavir (LPV/r)-based antiretroviral therapy (ART) in single dose nevirapine (sdNVP)-exposed HIV-infected infants: preliminary results from the IMPAACT P1060 trial. International AIDS Society, Cape Town, South Africa, 2009,

51. Kesho Bora Study Group. ThLBB105: Impact of Triple-ARV prophylaxis during pregnancy and breastfeeding compared with short-ARV prophylaxis for MTCT prevention on maternal disease progression. International AIDS Society, Vienna, Austria, 2010, http://pag.aids2010.org/Abstracts.aspx?SID=644&AID=17446.

52. Wright JC, Weinstein M. Gains in life expectancy from medical interventions--standardizing data on outcomes. *N Engl J Med* 1998,339:380-386.

53. Lawn SD, Myer L, Orrell C*, et al.* Early mortality among adults accessing a community-based antiretroviral service in South Africa: implications for programme design. *AIDS* 2005,19:2141-2148.

54. Yazdanpanah Y, Losina E, Anglaret X*, et al.* Clinical impact and cost-effectiveness of co-trimoxazole prophylaxis in patients with HIV/AIDS in Côte d'Ivoire: a trial-based analysis. *AIDS* 2005,19:1299-1308.

55. Anglaret X, Chene G, Attia A*, et al.* Early chemoprophylaxis with trimethoprim-sulphamethoxazole for HIV-1-infected adults in Abidjan, Côte d'Ivoire: a randomised trial. Cotrimo-CI Study Group. *Lancet* 1999,353:1463-1468.

56. Tuboi SH, Brinkhof MW, Egger M*, et al.* Discordant responses to potent antiretroviral treatment in previously naive HIV-1-infected adults initiating treatment in resource-constrained countries: the antiretroviral therapy in low-income countries (ART-LINC) collaboration. *J Acquir Immune Defic Syndr* 2007,45:52-59.

57. Gallant JE, DeJesus E, Arribas JR*, et al.* Tenofovir DF, emtricitabine, and efavirenz vs. zidovudine, lamivudine, and efavirenz for HIV. *N Engl J Med* 2006,354:251-260.

58. Johnson M, Grinsztejn G, Rodriguez C. Atazanavir plus ritonavir or saquinavir, and lopinavir/ritonavir in patients experiencing multiple virological failures. *AIDS* 2005,19:685-694.

59. Lockman S, Hughes MD, McIntyre J*, et al.* Antiretroviral therapies in women after single-dose nevirapine exposure. *N Engl J Med* 2010,363:1499-1509.

60. Murphy RA, Sunpath H, Lu Z*, et al.* Outcomes after virologic failure of first-line ART in South Africa. *AIDS* 2010,24:1007-1012.

61. United Nations. World Population Prospects: The 2008 Revision. 2009. Accessed on February 21, 2011 at http://esa.un.org/unpd/wpp2008/index.htm.

62. Marston M, Zaba B, Salomon JA*, et al.* Estimating the net effect of HIV on child mortality in African populations affected by generalized HIV epidemics. *J Acquir Immune Defic Syndr* 2005,38:219-227.

63. Gupte N, Bollinger R, Gupta A, for the SWEN India Study Team,. WEPEA110: High infant mortality rate among in-utero HIV-infected infants compared to postnatal mother-to-child HIV transmissions (MTCT) - survival benefit if early HIV transmission can be prevented International AIDS Society, Cape Town, South Africa, 2009,

64. Chilongozi D, Wang L, Brown L*, et al.* Morbidity and mortality among a cohort of human immunodeficiency virus type 1-infected and uninfected pregnant women and their infants from Malawi, Zambia, and Tanzania. *Pediatr Infect Dis J* 2008,27:808-814.

65. Spira R, Lepage P, Msellati P*, et al.* Natural history of human immunodeficiency virus type 1 infection in children: a five-year prospective study in Rwanda. Mother-to-Child HIV-1 Transmission Study Group. *Pediatrics* 1999,104:e56.

66. Fawzi WW, Msamanga GI, Hunter D*, et al.* Randomized trial of vitamin supplements in relation to transmission of HIV-1 through breastfeeding and early child mortality. *AIDS* 2002,16:1935-1944.

67. Leroy V, Karon JM, Alioum A*, et al.* Twenty-four month efficacy of a maternal short-course zidovudine regimen to prevent mother-to-child transmission of HIV-1 in West Africa. *AIDS* 2002,16:631-641.

68. Moodley D, Moodley J, Coovadia H*, et al.* A multicenter randomized controlled trial of nevirapine versus a combination of zidovudine and lamivudine to reduce intrapartum and early postpartum mother-to-child transmission of human immunodeficiency virus type 1. *J Infect Dis* 2003,187:725-735.

69. Thistle P, Gottesman M, Pilon R*, et al.* A randomized control trial of an Ultra-Short zidovudine regimen in the prevention of perinatal HIV transmission in rural Zimbabwe. *Cent Afr J Med* 2004,50:79-84.

70. Kiarie JN, Kreiss JK, Richardson BA, John-Stewart GC. Compliance with antiretroviral regimens to prevent perinatal HIV-1 transmission in Kenya. *AIDS* 2003,17:65-71.

71. Taha TE, Kumwenda NI, Hoover DR*, et al.* Nevirapine and zidovudine at birth to reduce perinatal transmission of HIV in an African setting: a randomized controlled trial. *JAMA* 2004,292:202-209.

72. Kumwenda NI, Hoover DR, Mofenson LM*, et al.* Extended antiretroviral prophylaxis to reduce breast-milk HIV-1 transmission. *N Engl J Med* 2008,359:119-129.

73. Coovadia HM, Rollins NC, Bland RM*, et al.* Mother-to-child transmission of HIV-1 infection during exclusive breastfeeding in the first 6 months of life: an intervention cohort study. *Lancet* 2007,369:1107-1116.

74. Connor EM, Sperling RS, Gelber R*, et al.* Reduction of maternal-infant transmission of human immunodeficiency virus type 1 with zidovudine treatment. Pediatric AIDS Clinical Trials Group Protocol 076 Study Group. *N Engl J Med* 1994,331:1173-1180.

75. Chasela C, Hudgens M, Jamieson D*, et al.* Both maternal HAART and daily infant nevirapine (NVP) are effective in reducing HIV-1 transmission during breastfeeding in a randomized trial in Malawi: 28 week results of the Breastfeeding, Antiretroviral and Nutrition (BAN) Study 5th IAS Conference on HIV Pathogenesis, Treatment and Prevention, Cape Town, South Africa, 2009,

76. Bedri A, Gudetta B, Isehak A*, et al.* Extended-dose nevirapine to 6 weeks of age for infants to prevent HIV transmission via breastfeeding in Ethiopia, India, and Uganda: an analysis of three randomised controlled trials. *Lancet* 2008,372:300-313.

77. Kesho Bora Study Group. Eighteen-month follow-up of HIV-1-infected mothers and their children enrolled in the Kesho Bora study observational cohorts. *J Acquir Immune Defic Syndr* 2010,54:533-541.

78. Peltier CA, Ndayisaba GF, Lepage P*, et al.* Breastfeeding with maternal antiretroviral therapy or formula feeding to prevent HIV postnatal mother-to-child transmission in Rwanda. *AIDS* 2009,23:2415-2423.

79. World Health Organization. Antiretroviral drugs for treating pregnant women and preventing HIV infection in infants: towards universal access. 2010.Accessed on February 21, 2011 at http://whqlibdoc.who.int/publications/2010/9789241599818_eng.pdf.

80. Carter RJ, Dugan K, El-Sadr WM*, et al.* CD4+ Cell Count Testing More Effective Than HIV Disease Clinical Staging in Identifying Pregnant and Postpartum Women Eligible for Antiretroviral Therapy in Resource-Limited Settings. *J Acquir Immune Defic Syndr* 2010,55:404-410.
